# Impact of indispensable amino acid supplementation on gut function in children at high risk of environmental enteropathy: protocol for an international coordinated group of randomised controlled trials

**DOI:** 10.1136/bmjopen-2025-105456

**Published:** 2026-04-24

**Authors:** Gwenyth O Lee, Victor Owino, Amster Fei P Baquiran, Roshini M Pasanna, Seyram Elom Achoribo, Toufik Meskini, Beatrice Amadi, Kenneth Mphatso Maleta, Claire Gaudichon, Michael E Serafico, Shalini Hegde, Carl Vincent D Cabanilla, Sarita Devi, Mohammed El Mzibri, Andrew F Brouwer, Anura V Kurpad, Paul Kelly, Douglas Morrison

**Affiliations:** 1 Department of Biostatistics and Epidemiology, School of Public Health, Rutgers The State University of New Jersey, Piscataway, New Jersey, USA; 2 Rutgers Global Health Institute, Rutgers The State University of New Jersey, New Brunswick, New Jersey, USA; 3 Nutritional and Health Related Environmental Studies Section, Division of Human Health, International Atomic Energy Agency, Vienna, Austria; 4 Department of Science and Technology-Food and Nutrition Research Institute, Taguig City, Philippines; 5 Division of Nutrition, St John’s Research Institute, St John’s National Academy of Health Sciences (a Unit of CBCI Society for Medical Education), Bangalore, Karnataka, India; 6 Radiological and Medical Sciences Institute, Ghana Atomic Energy Commission, Kwabenya-Accra, Ghana; 7 Nutrition Gastroenterology Metabolic Disease (NUTGAMED) research structure, Mohammed V University in Rabat, Rabat, Morocco; 8 Tropical Gastroenterology and Nutrition Group, Department of Medicine, University of Zambia School of Medicine, Lusaka, Zambia; 9 Department of Nutrition and Dietetics, School of Global and Public Health, Kamuzu University of Health Sciences, Blantyre, Malawi; 10 INRAE, UMR PNCA, Université Paris-Saclay AgroParisTech, Paris, France; 11 Department of Pediatric Surgery, St John's Medical College Hospital, St John's National Academy of Health Sciences (A unit of CBCI Society for Medical Education), Bangalore, Karnataka, India; 12 Life Sciences Division, National Centre of Energy, Nuclear Sciences and Technics (CNESTEN), Rabat, Morocco; 13 Department of Epidemiology, University of Michigan, Ann Arbor, Michigan, USA; 14 Blizard Institute, Barts and The London School of Medicine, Queen Mary University of London, London, UK; 15 Scottish Universities Environmental Research Centre (SUERC), University of Glasgow, Glasgow, UK

**Keywords:** Clinical Trial, Gastrointestinal infections, Malabsorption, Community child health, Nutrition

## Abstract

**Introduction:**

Environmental enteropathy (EE) is a syndrome affecting the gut characterised by villus blunting, reduced nutrient absorption and microbial translocation in children and adults experiencing a high burden of enteric infection due to inadequate access to clean water and sanitation.

**Methods and analysis:**

We will conduct coordinated randomised controlled trials in six countries to determine if supplementation with indispensable amino acids (IAAs) can improve intestinal barrier dysfunction in six geographically diverse populations of 18–36 months old children with stunting or severe stunting. All trials will measure the same primary outcomes while secondary outcomes will be measured on a per-trial basis using standardised protocols across the project. The primary endpoint will be change in gut permeability as assessed by the lactulose/rhamnose ratio. Secondary endpoints include changes in amino acid and carbohydrate absorption using novel, isotope tracer tests. Other prespecified outcome measures include changes in EE biomarkers and child weight. IAA supplementation will be given daily for 28 days and evaluation of the major endpoints will be at baseline and after 28 days of supplementation.

**Ethics and dissemination:**

Ethical approval will be obtained from the Research Ethics Committee at each participating site. Caregivers will provide written informed consent for each participant. Findings will be disseminated through peer-reviewed journals, conference presentations and face-to-face meetings with participant caregivers.

**Trial registration number:**

CTRI: CTRI/2024/06/069187 (India); ClinicalTrials.gov (NCT06617130, Malawi; NCT06676215, Philippines and NCT07256028, Morocco); Ongoing (Zambia); Ongoing (Morocco); PACTR: (PACTR202311714091884, Ghana).

**STRENGTHS AND LIMITATIONS OF THIS STUDY**

- Our study aims to better understand whether amino acid supplementation can promote gut health among stunted children, a question of global public health importance.
- Our coordinated study approach will enrol children from diverse low- and middle-income country settings.
- We use stable isotope-based tests to directly assess protein digestibility (using the dual stable isotope test) and carbohydrate absorption (using the ^13^C sucrose breath test).
- We are limited in our inability to examine long-term outcomes, such as improvements in linear growth associated with the intervention.

## Introduction

 Approximately 20% of children under 5 years worldwide experience chronic growth faltering,^1^ with lifelong consequences for health and well-being.^2^ While the causes of growth faltering are understood to be multifactorial,^3^ environmental enteropathy (EE), a condition defined by alterations in small intestinal structure and function,^4^ is an important risk factor for growth faltering in infancy.^5^,^7^

**Figure 1:**
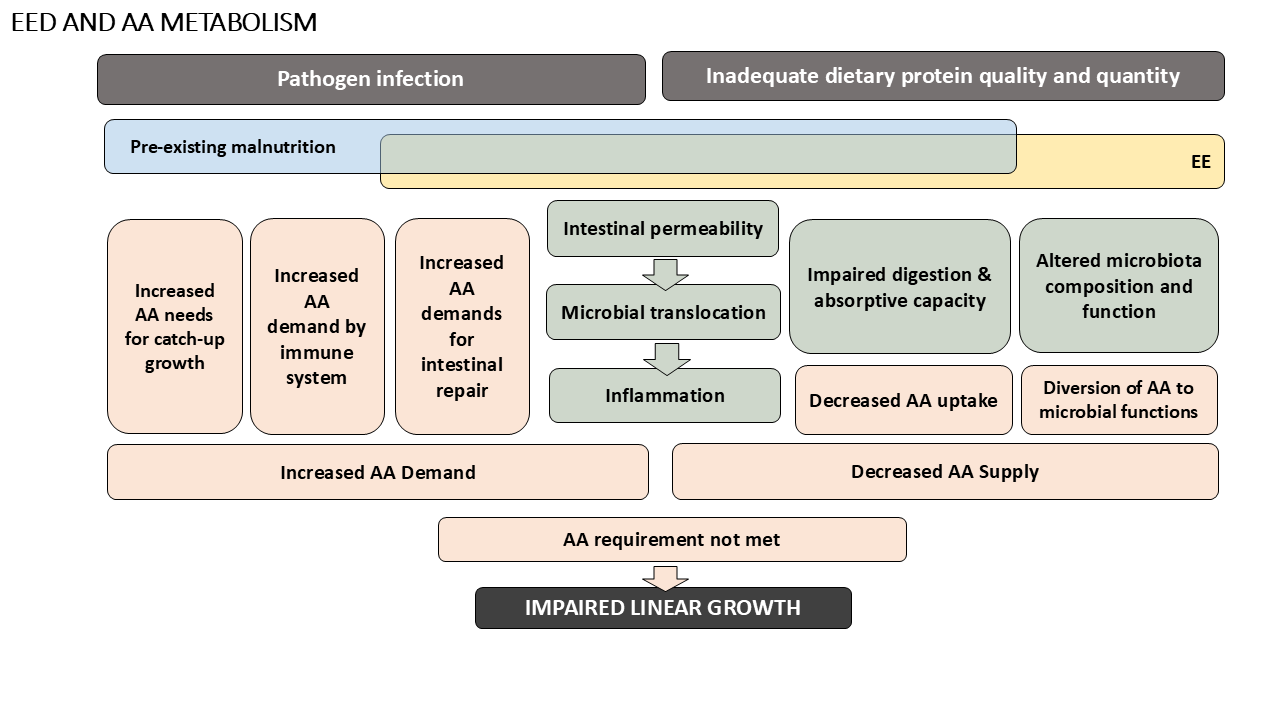
Hypothesised relationships between EE, AA requirements and linear growth faltering. Hypothesised relationships related to how EE may impact AA requirements for children at risk of growth faltering. AA, amino acid; EE, environmental enteropathy.

EE results from frequent exposure to enteric pathogens, especially in the context of inadequate or marginally adequate dietary protein intake^8^,^10^ and leads to increased intestinal inflammation, alterations to the gut microbiota and reductions in nutrient absorptive capacity in the small intestine.^11^ EE may also increase requirements for nutrients related to intestinal immune responses and recovery, especially high-quality dietary protein.^11^ Given that protein is required to provide indispensable amino acids (IAAs) for protein biosynthesis and normal growth in children,^12^ it has been hypothesised that the growth faltering observed in EE may result from depleted IAA status.^13^ There is some evidence to support this hypothesis from animal studies^13^; and lower circulating plasma concentration of some amino acids (eg, plasma citrulline and tryptophan) have been associated both with biomarkers of EE (urinary lactulose/rhamnose (LR) ratio)^14^ and linear growth faltering in children^15^ (figure 1).

Improving amino acid status may also promote recovery from EE, because amino acids have important benefits for gut health.^11^ Amino acids are both metabolised in enterocytes to support their high energy demands and used as building blocks for bioactive compounds required for immune response, epithelial renewal and mucin production.^16^ As a result, it has been proposed that nutritional interventions to prevent or optimise recovery from EE should provide IAAs to ‘replenish depleted stores’^11^ and improve gut health. Several studies have examined the impact of amino acid supplementation on EE biomarkers, primarily in adults, although with considerable variation in the amino acid supplement mix and with varying results.^17 18^ While most of these studies used the dual-sugar test (urinary lactulose/mannitol (LM) or LR ratio) as an outcome, one study in Zambian adults examined the gut directly and observed that tryptophan, leucine and glutamate supplementation protected against seasonal villus height reduction.^17^

An additional difficulty in EE research is that, although EE is hypothesised to impact the gut through functional changes to nutrient absorption, in many cases only limited measures of gut status (eg, inflammatory biomarkers) are assessed. The LM or LR ratio is a commonly used functional test for EE, but poses challenges in both administration and interpretation.^19^ To overcome this obstacle, stable isotope tracer tests have been developed to measure carbohydrate^20^ and protein digestion and absorption.^21^ The ^13^C sucrose breath test (^13^C-SBT) assesses intestinal sucrase-isomaltase activity, which may be suppressed in EED.^22^ The dual stable isotope test (DSIT) measures the digestion and absorption of protein-derived amino acids, making it especially valuable for understanding protein metabolism in children with EE or stunting. One study of Indian children using the DSIT found no statistically significant association between EE and amino acid absorption.^23^ Another study conducted in Pakistan hypothesised that children with EE may have compensatory/adaptive upregulation of enzymes associated with protein transport in the gut,^24^ which may help to explain these findings.

In this protocol, we describe a coordinated research project that aims to evaluate the impact of 28 days of IAA supplementation on EE among children with growth faltering in six low-resource settings.

### Hypothesis

IAA supplementation will improve biomarkers of gut function and functional measures of carbohydrate and protein absorption among children with stunting and at high risk of EE.

### Study objectives

The objectives of this study are to determine the effects of IAA supplementation for 28 days on:

1. The dual sugar LR ratio test.
2. Secondary outcomes, including (1) other biomarkers of EE (plasma intestinal fatty acid binding protein (iFABP), plasma lipopolysaccharide binding protein (LPSBP), faecal myeloperoxidase (MPO) and faecal neopterin (NEO), (2) amino acid digestion, assessed by a DSIT, (3) carbohydrate digestion assessed by ^13^C-SBT and (4) weight gain.

## Methods and analysis

### Overview of the trial design

**Figure 2:**
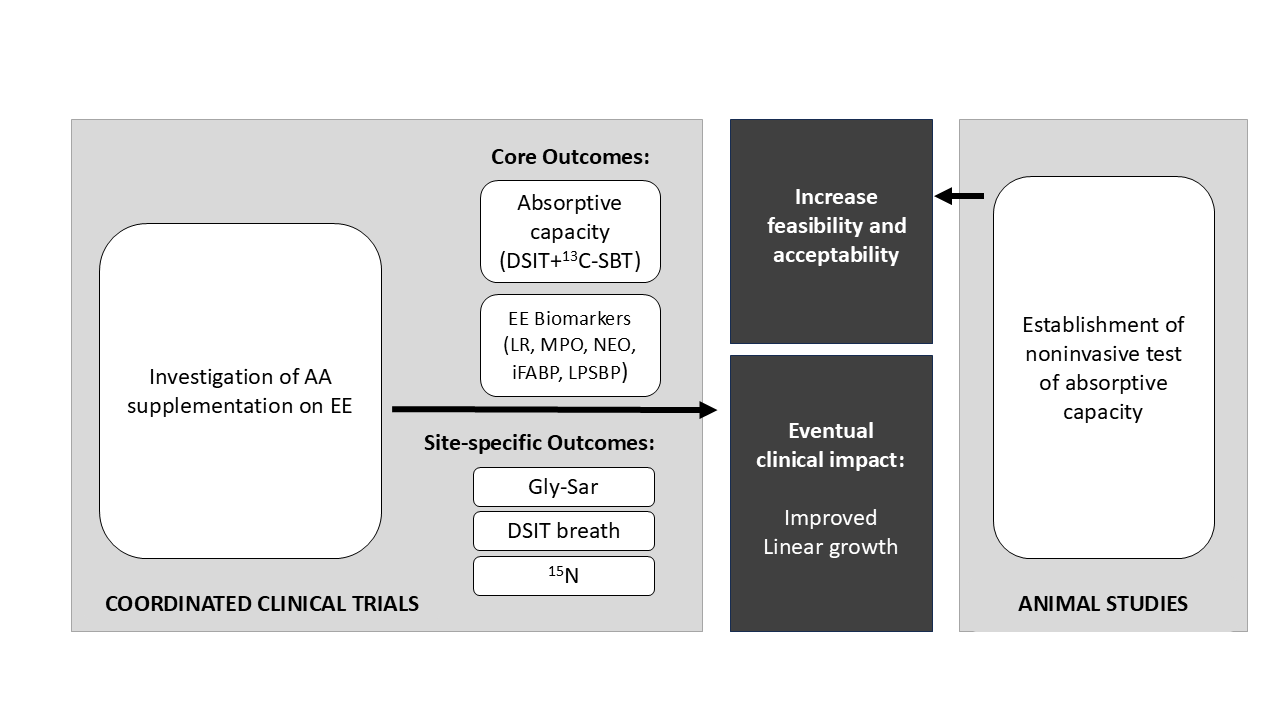
Coordinated research project design. A visual depiction of our coordinated research project design. AA, amino acid; 13C-SBT, 13C sucrose breath test; DSIT, dual stable isotope test; EE, environmental enteropathy; Gly-Sar, glycylsarcosine; iFABP, intestinal fatty acid binding protein; LPSBP, lipopolysaccharide binding protein; LR, lactulose/rhamnose test; MPO, faecal myeloperoxidase; NEO, faecal neopterin.

We will conduct a group of six coordinated, randomised controlled trials (RCTs). We opted for coordinated trials instead of a single, multisite trial to adhere to local regulatory and administrative guidelines and accommodate the logistical capacities and epidemiological context of each site. Each site will adhere to a core protocol, with variation in recruitment procedures (eg, recruitment through community, daycare or well-child visits) and room for additional outcome measures according to each site’s capacity (see online supplement 1 for full methodological details for each site). These trials will be conducted within the context of a larger, coordinated research programme that also includes complementary animal studies in France. In this protocol paper, we describe only the coordinated RCTs (figure 2) in six countries namely, Ghana, India, Malawi, Morocco, the Philippines and Zambia.

**Figure 3:**
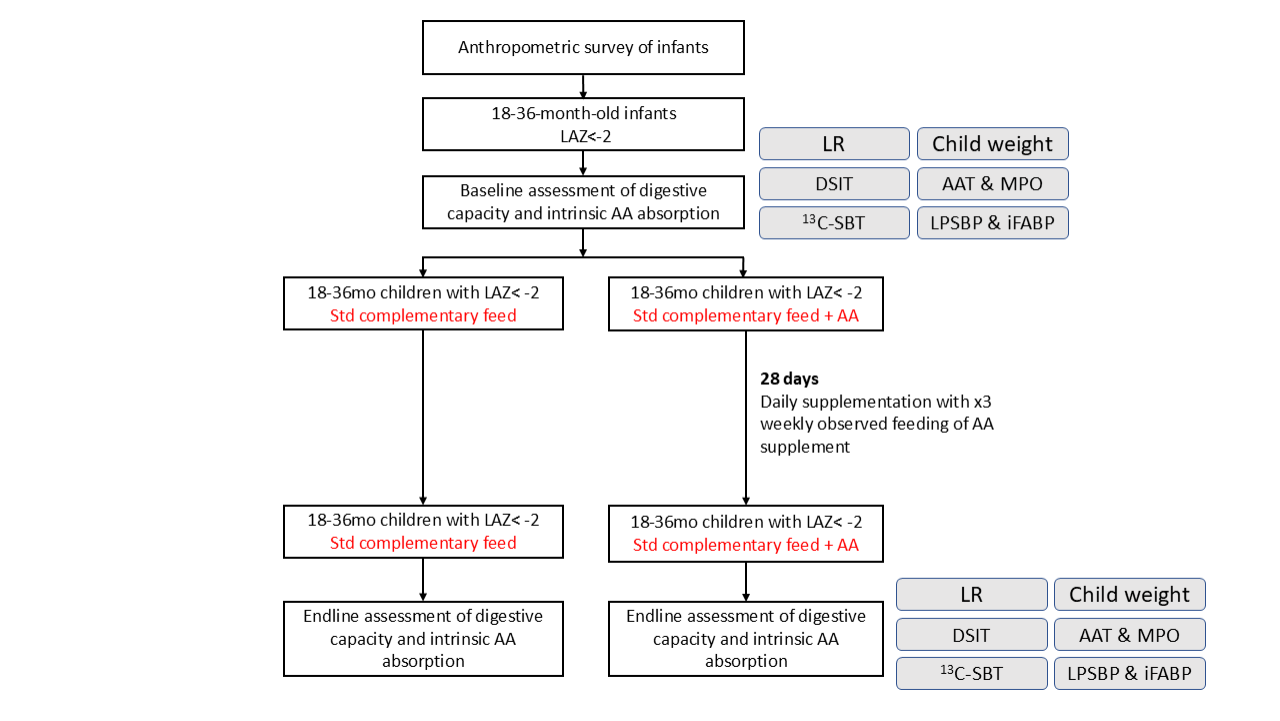
Study flow chart. This figure shows the complete list of primary (LR) and secondary (DSIT, 13C-SBT, AAT, MPO, LPSBP and iFABP) biomarkers planned across the trials. Not all trials will administer all outcome measures. AA, amino acid; AAT, alpha-1 antitrypsin; 13C-SBT, 13C sucrose breath test; DSIT, dual stable isotope test; iFABP, intestinal fatty acid binding protein; LAZ, length-for-age z-score; LPSBP, lipopolysaccharide binding protein; LR, lactulose/rhamnose; mo, month old; MPO, faecal myeloperoxidase.

This is an open label study. In each trial, 18–36 months old children in the intervention arm will receive IAA supplementation for 28 days, while children in the control arm will receive only standard complementary foods for the same duration (figure 3). The study will be unblinded. Each of the six study sites will aim to recruit 60 children, half in the intervention arm and half in the control arm (see ‘Sample Size section’ for further details). Data collection began in August 2025 and is anticipated to be completed in all sites by June 2026. Site-specific start dates and enrolment status are reported in online supplement 1 (Standard Protocol Items: Recommendations for Interventional Trials (SPIRIT) guidelines for each site).

### Data collection and participant timeline

**Figure 4:**
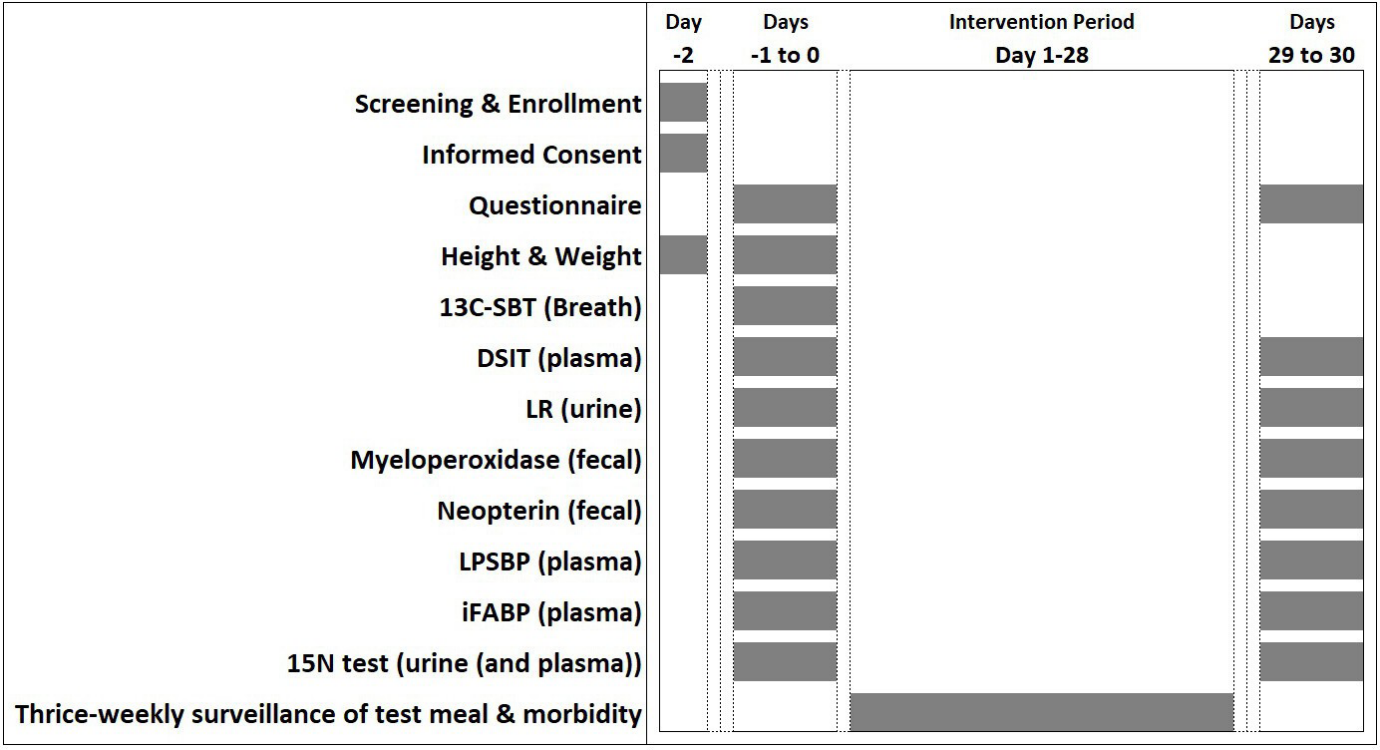
Protocol timeline. This figure shows the complete list of primary (LR) and secondary (DSIT, 13C-SBT, AAT, MPO, LPSBP and iFABP) biomarkers planned across the trials. Not all trials will administer all outcome measures. AAT, alpha-1 antitrypsin; 13C-SBT, 13C sucrose breath test; DSIT, dual stable isotope test; iFABP, intestinal fatty acid binding protein; LPSBP, lipopolysaccharide binding protein; LR, lactulose/rhamnose; MPO, faecal myeloperoxidase.

The overall timeline for each participant in the studies is visualised in figure 4. Each study site will follow their own preferred protocol for recruitment and enrolment. Following enrolment, baseline study procedures will be completed over two visits. Each participating child will then be provided with amino acid supplementation at the daily dosage described in table 1, for 28 days. End-line data will again be collected over two visits, with flexibility for scheduling based on the outcome measures each site intends to collect and logistical preferences (table 2). Each site will manage data entry, data storage and other data management procedures in accordance with local regulations and institutional guidance. A detailed description of the study intervention and aspects of the specific outcome tests and measurements to be performed at baseline and endline are described in figure 4 and further detailed below:

### Ethics declaration

**Table 1:** A representative total dose of the IAA supplement in milligrams per day, calculated for a 24-month-old child

IAA	Dose
Gly	232.1 mg/day
His	252 mg/day
Ile	453.6 mg/day
Leu	907.2 mg/day
Lys	739.2 mg/day
Met	246.3 mg/day
Cys	123.1 mg/day
Phe	336.0 mg/day
Tyr	336.0 mg/day
Thr	403.2 mg/day
Trp	100.8 mg/day
Val	604.8 mg/day
Total	4734.5 mg/day

Gly is added to support intestinal glutathione synthesis.^54^ The Gly dose is calculated as 63% of AA intake using whole egg protein as a reference.

AA, amino acid; Cys, cysteine; Gly, glycine; His, histidine; IAA, indispensable amino acid; Ile, isoleucine; Leu, leucine; Lys, lysine; Met, methionine; Phe, phenylalanine; Thr, threonine; Trp, tryptophan; Tyr, tyrosine; Val, valine.

**Table 2:** Planned outcome measures in each site

	Ghana	India	Malawi	Morocco	Philippines	Zambia
LR	5-hour	5-hour	5-hour	3-hour	5-hour	3-hour
DSIT	Yes	Yes	Yes	Yes	Yes	No
13C-SBT	Yes	Yes	Yes	Yes	Yes	No
Faecal MPO and NEO	Yes	Yes	Yes	Yes	Yes	Yes
LPSBP and iFABP	Yes	Yes	Yes	Yes	Yes	Yes

13C-SBT, 13C sucrose breath test; DSIT, dual stable isotope test; iFABP, intestinal fatty acid binding protein; LPSBP, lipopolysaccharide binding protein; LR, lactulose/rhamnose; MPO, myeloperoxidase; NEO, neopterin.

Ethical approval for the study has been obtained from the Ethical Review Board overseeing research at each site. The research protocol follows ‘the Standard Protocol Items: Recommendations for Interventional Trials (SPIRIT)’.^25^

### Study setting and participants

This study has been or will be conducted in six settings: Accra, Ghana (Ghana Atomic Energy Commission); Bangalore, India (St John’s Research Institute); Taguig, Philippines (Food and Nutrition Research Institute); Blantyre, Malawi (Kamuzu University of Health Science), Rabat, Morocco (National Center for Energy, Nuclear Sciences and Techniques) and Lusaka, Zambia (TROPGAN and the University of Zambia). This multicountry coordinated study design supports our capacity to capture important variation in the aetiology of stunting. For example, the relative importance of inadequate diet quality versus specific enteric pathogen exposures may vary by context (table 3). A high burden of enteric pathogen infections in early life^26^,^28^ may impact small intestinal function^29^ in a manner that may increase protein and AA requirements even though infant diets meet standard recommended protein intakes.^30^

**Table 3:** Site characteristics

	Ghana*	India†	Malawi†	Morocco‡	Philippines§	Zambia†
Rural/urban	Urban	Urban	Rural	Urban	Urban	Urban
Study setting	Hospital	Hospital	Health centre	Hospital	Community	Community
Under five stunting prevalence	18%	32.3%	42%	15.5%	23.6%	35%
Under five wasting	6%	6.3%	3%	3%	5.6%	4%
Food insecurity prevalence	3%	58.1%	60%	36%	31.4%	93.4%
Minimum dietary diversity—prevalence	13%	51.6%	15%	60%	13.8%	42.4%

* Based on the Ghana demographic health surveys^55^ and statistics reported by the FAO^56^ and UNICEF.^57^

† As reported in prior studies^58^ or from unpublished data.

‡ As reported by World Bank country income classification; Human Development Index Score and Global Hunger Index severity level and by Moroccan nutrition survey (2019–2020).

§ 2023 National Nutrition Survey for stunting, wasting and food insecurity data and 2021 National Nutrition Survey for minimum dietary diversity data.

FAO, Food and Agriculture Organization.

### Eligibility criteria

Participants must meet all the inclusion and exclusion criteria to be enrolled in the study (table 4). At each site, study treatment will not begin until a participant is enrolled.

**Table 4:** Inclusion and exclusion criteria

Inclusion criteria	Exclusion criteria
Of either sex	
18–36 months old	<18 months old >36 months old
Stunted (LAZ <−2) or severe stunting (LAZ <−3) (in Zambia)	Wasted (WLZ <−2) Overweight (WLZ >2)
	Moderate and severe anaemia (Hb≤99 g/L) (in Malawi)
For African sites, able and willing to undergo HIV testing	Have any underlying condition other than HIV, which in the opinion of the investigator would put the participant at undue risk of failing study completion or would interfere with analysis of study results.
Parent, caregiver or guardian able and willing to provide written informed consent	
	Have had diarrhoea (by self-report) in the preceding month
	Sibling or twin of another participating study child (two children of different families in the same household are both eligible)

Hb, haemoglobin; LAZ, length-for-age z-score; WLZ, weight-for-length z-score.

### Randomisation

Following enrolment, participants will be assigned into either the intervention or control arm of the study with equal probability. Block randomisation will be employed to ensure comparable allocation in the sequence of assignments of the participant. The randomisation sequence generation and allocation for the trial will be conducted by the coordinating team at Rutgers University using a random number generator with a fixed reproducible seed. Randomisation data will be provided to study sites using opaque, sealed envelopes.

### Study intervention

Following the baseline study assessments, each participant in the intervention group will receive a daily IAA supplement, to be mixed into the child’s usual complementary food. The crystalline IAA mix powder (containing histidine, isoleucine, leucine, lysine, methionine, phenylalanine, threonine, tryptophan and valine).

The daily dose provides ×1.5 times the daily estimated average requirement (EAR) for a healthy child. This dose was estimated as follows. First, children with EE are likely to have higher protein requirements than healthy children: although no direct data on children with EE is available, it has been estimated that protein requirements are increased by 10% in children with intestinal parasites, 20% in children convalescing from acute bacterial infections and 30% in those recovering from acute diarrhoea.^31^ Second, based on the WHO/Food and Agriculture Organization 2007 recommendations, a safe level of protein intake is set at +1.96 SD above the EAR^31^: equivalent to 1.22 times the EAR. Therefore, the study dose is 1.22 times the EAR, multiplied by 1.25 to reflect an estimated ∼25% increase in protein requirements for a child with EE, resulting in a total of 1.5 times the EAR. This IAA dose will be taken once daily by the child for 4 weeks (28 days). The study dose is calculated according to the weight of the child. The IAA supplement was prepared in a single batch and distributed to the six sites in convenient heat-sealed, moisture resistant packaging for dispensing on site. Further details of the IAA mix quality specifications, producer and quality assurance systems may be found in the supplemental materials (online supplement 2, Detailed description of intervention).

#### Intervention monitoring

The study team will directly observe up to three feedings per week (12 out of the 28 days period). The uptake on non-observed days will be based on the caregiver’s report. On each intervention monitoring day, a questionnaire will also be administered to assess child morbidity for that day and on the day prior.

#### Safety issues for intervention

There is currently no established upper limit for safe amino acid intake.^32^ However, there are some reports of adverse effects associated with high dosages (∼10–30 times higher than proposed in this study), especially with prolonged use.^33^ The doses we propose are similar to those used in multiple prior studies to promote growth and development in low birth weight or premature infants.^34^ A recent systematic review noted no increase in the incidence of adverse events in these trials.^34^

#### Outcomes and case definitions

We will collect child stool, blood, urine and breath samples, both at baseline and endline, to conduct the following tests of intestinal status and function. All outcome measures for this study are expressed as changes from baseline.

#### Primary outcomes

*LR dual-sugar test*. The LR test is the most commonly used method to assess intestinal disruption in EE, because it is posited to measure intestinal permeability.^35^ At baseline and endline, the LR test will be administered. Guardians will be asked to fast their child for 1 hour prior to the appointment. A single urine sample will be collected before the start of the study and the time noted. At the start of the test, a 9 mL of drinking water spiked with 1 g lactulose and 0.2 g rhamnose will be administered, and again a 2 mL chaser of drinking water will be used to rinse the vial. The total volume (1 mL sucrose solution+2 mL chaser+9 mL LR solution+2 mL chaser) is 14 mL. All urine passed from 30 min up to 5 hours will be collected and pooled and the total volume noted. Water or breastmilk can be freely consumed throughout the test period. Sites have chosen between a possible 3-hour or a 5-hour LR collection protocols based on local logistical considerations (ie, ease of administration in young children).

#### Secondary outcomes

*DSIT of protein digestion and absorption*. The DSIT will be administered to test the extent to which protein derived amino acids present in the test meal are released into circulation. The DSIT has previously been described elsewhere.^21 23^ In brief, complete digestion and absorption of protein derived amino acids will equate to the ratio of ^13^C amino acids in protein to ^2^H free amino acids in the meal being equal to the isotopologues ratio in plasma amino acids. Deviation from equality of amino isotopologue ratios between meal and plasma pools implies impaired gut function and reduced bioavailability of protein derived amino acids. To complete the DSIT, a single blood sample is collected from the child before the start of the study. The child will then receive a test meal designed to provide 1/3 of their daily calorie requirements as well as a dual stable isotope tracer mix of U-^13^C spirulina (10 mg/kg body weight) and ^2^H-AA mix (1.25 mg/kg body weight). The test meal is split into nine mini-meal portions. The child will first consume a three-portion priming meal followed by five portions consumed hourly to achieve a plateau in AA plasma isotopic enrichment. The ninth mini-meal portion is frozen to −80°C degrees, lyophilised, ground and stored for later analysis. Water or milk can be freely consumed throughout the test period. An additional blood sample will be collected at 5 hours after the commencement of the study in a simplified protocol based on a previous study that shows plateauing of isotopic enrichments at 5 hours.^21^

*^13^C-SBT*. The ^13^C-SBT is a breath test used to assess intestinal sucrase-isomaltase activity, which may be a proxy for alterations to intestinal function in.^2036^,^38^ A baseline breath sample is collected before giving the child a 40 mg/mL ^13^C_12_-sucrose solution, administered at a dosage of 10 µL per kg body weight (spiked in 1 mL of drinking water). This is followed by breath sample collections every 15 min for the first hour and then every 30 min for another 3 hours (a total of 4 hours). The child should consume only water or milk over the first 90 mins of the test where possible.

*Plasma biomarkers of gut function*. Plasma samples taken during the DSIT will also be used to assess *LPSBP* and iFABP, biomarkers that are elevated in EE.

*Faecal MPO and NEO*. Faecal samples will be collected to assess faecal MPO and NEO, frequently-used measures of intestinal inflammation in EE.^5^ NEO is a marker of interferon-gamma mediated activation of macrophages by T helper cell 1,^39 40^ while MPO is largely a marker of neutrophil activation.^5^

#### Additional outcomes

Questionnaires will be used to collect demographic (eg, age, sex, race) and socio-economic information using the Water, Assets, Maternal education and Income index.^41^ In addition, questionnaires will be used to assess dietary data (dietary diversity), modified to include information about the consumption of plant foods that fix CO via a C_4_ (Hatch and Slack) pathway, resulting in a greater natural abundance of ^13^C that may elevate baseline ^13^CO_2_ levels in the breath in participants.^42^

#### Additional optional outcomes

Our coordinated clinical trial design also implies that each trial team has flexibility in including additional optional outcomes, dependent on the capacity and scientific interests of the team. Optional outcomes include the following:

*Breath sampling during the DSIT*. To evaluate whether ^13^CO_2_ breath testing during the DSIT can be used as a non-invasive test of protein digestion, a priming dose of NaH^13^CO_3_ (3 µmol/kg) will be added to the DSIT meal. We will perform a correlative study of ^13^CO_2_ breath at plateau enrichment, that is, parts per million (ppm) of ^13^C post dosing minus baseline ppm ^13^CO_2_, in comparison to the DSIT result.

*^15^N test to measure dietary N bioavailability during the DSIT*. To evaluate whether the AA supplementation improves dietary nitrogen bioavailability, the DSIT 3-portion priming meal may also be extrinsically labelled with commercially affordable 98% enriched ^15^N spirulina (5 mg/kg). The ^15^N recovery will be followed in plasma and urinary metabolic pools (using the same samples collected to assay the primary and secondary study outcomes) that are known to be sensitive to amino acid absorption. Indeed, it was reported in pigs^43^ and humans^44^ that protein malabsorption induced by chronic pancreatitis was associated with a lower postprandial recovery of ^15^N in plasma protein and urinary urea.

*Glycylsarcosine (Gly-Sar) test*. Amino acids can be absorbed in small peptide form as well as free amino acids. To evaluate gut dipeptide by the transporter PepT1, Gly-Sar^45^ may be administered alongside the DSIT and/or LR tests. A single urine sample is collected before the start of the test, followed by a 1 g dose of Gly-Sar administered with 10 mL water. All urine passed up to 5 hours is collected and pooled and the total volume noted.

### Safety issues for outcome measures

This study uses a combination of common and newer tests to assess infant gut function. Some of these involve providing the infant with a small dose of tracer molecules to estimate intestinal absorption of the probe. These tests are: the dual sugar LR test of intestinal permeability,^35^ the ^13^C-SBT of intestinal sucrase activity^20 38^ and the DSIT of protein absorption.^23^ The LR test involves feeding infants a small dose of sugary solution, the ^13^C-SBT similarly involves a small dose of ^13^C-labelled sucrose solution and the DSIT involves feeding infants U-^13^C spirulina, ^2^H-AA mix and NaH^13^CO_3_ mixed into complementary foods. Stable isotopes are not radioactive and are naturally found in food sources,^46^ so the risks associated with the ^13^C-SBT and DSIT are equivalent to those of consuming the unlabelled probe. One health risk associated with these tests is that some infants may not tolerate the solution (eg, the sugary solution may cause them to vomit or spit up) or they may not like the unfamiliar taste of spirulina, a plant-based protein with a bright blue-green colour. An additional health risk of the DSIT is that it requires two 2 mL blood collections over a 5-hour period and an additional 0.5 mL for the inflammatory marker tests (LPSBP and iFABP). The two-point blood collection is currently the minimal acceptable protocol to produce interpretable data on specific amino acid absorption. The LR test requires non-invasive urine collection, the ^13^C-SBT requires non-invasive breath collection and MPO and NEO tests require faecal collection. The risk to the child is that they may be uncomfortable or not like having their urine/breath/faeces collected. Study staff will employ gentle, child-friendly methods to minimise distress.

### Biological sample collection and archiving

All biological samples will be collected, processed and preserved as per the study standard operating procedures (SOPs). Faecal samples will be collected and aliquoted into sterile, prelabelled 2 mL cryovials without additives. For blood samples, the study team will collect whole venous blood samples as per the SOPs. The sample will be centrifuged to separate plasma, and the plasma will then be aliquoted and stored at −80°C. Urine samples will be aliquoted and stored immediately at −80°C without preservative. Breath samples will be collected in white-capped, finger-tight breath collection tubes, stored at room temperature and sent to the laboratory no more than 3 months after the initial collection.

### Laboratory analysis

Laboratory analyses will be conducted either on site or at centralised laboratories, depending on the requirements of the test outcome measure. Faecal biomarkers and plasma iFABP and LPSBP will be assessed on site. Urine samples will be sent to St John’s Research Institute laboratory for L/R analysis by gas chromatography mass spectrometry (MS). Plasma samples and test meal food samples will also be sent to St John’s Research Institute or the University of Glasgow laboratory for the ^2^H/^13^C DSIT analysis by liquid chromatography MS/MS. Breath samples will be sent to St John’s Research Institute laboratory for ^13^C analysis by isotope ratio mass spectrometry (IRMS) or to the University of Glasgow. Urine (total volume to be recorded) and plasma samples from children receiving an additional dose of ^15^N spirulina will be sent to AgroParisTech to be measured by EA-IRMS.

### Data safety monitoring plan

Each trial site will appoint their own Data Safety and Monitoring Board, independent of the sponsor, according to local legal and regulatory requirements.

### Monitoring adverse events

Study participants will be monitored for any adverse events or significant changes in clinical status. If any adverse events are observed, participants will be referred for treatment. The specific protocol for referral and treatment is separately established by the local site. All adverse events will be reported to the appropriate ethics review board and study registry.

### Sample size

The sample size for this study is based on the minimum number of participants necessary to observe differences in the primary outcome variable (ΔLR) between intervention and control groups.^47 48^ Each site will contribute data from 60 children, for a total of 360 children across six sites. Assuming an intraclass correlation coefficient (ICC) of 0.1 in ΔLR ratios based on prior reports,^49^ this is sufficient to detect a standardised effect size of 0.40 or greater (a change in ΔLR equal to or greater than 0.4 SD) with an α value of 5% and 80% power. This is equivalent to changes in LR reported in response to other nutritional interventions^47 48^ and is substantially greater than previously reported effect sizes for glutamine and glycine.^50^ A sample size of 60 participants is also sufficient to detect within-site differences with a standardised effect size of 0.84 or greater (ΔLR equal to or greater than 0.84 SD). This difference is on the order of the prior reports of glutamine and glycine supplementation.^50^ Assuming a similar ICC for DSIT results, the same effect sizes (0.40 or greater for pooled analyses and 0.84 or greater for within-site analyses) would be detectable. A prior study on specific amino acid absorption in children within and without EE reported differences in mean amino acid absorption between the group equivalent to an effect size of 0.5.^51^ However, our study will be the first to use the DSIT to evaluate changes in protein digestion associated with a nutritional intervention.

#### Data collection, management and storage

Data collection tools include survey instruments and results of the outcome tests described above. All participant data will be collected using ID codes to maintain confidentiality and enable the participant’s tracking throughout the study period. All laboratory specimens will also be labelled with sample ID codes.

#### Statistical analysis

To assess the effect of amino acid supplementation on markers of gut function in stunted children, we will first calculate summary values for the LR, ^13^C-SBT and the DSIT. Each of these tests may be summarised in various ways, and so our primary and secondary summary measures for each test are determined a priori. The LR test is the urinary excretion ratio (% of lactulose dose excreted/% of rhamnose dose excreted) over the test period; % lactulose excretion and % rhamnose excretion will be included as secondary outcomes. For the ^13^C-SBT, increases in atom per cent excess over baseline abundance will be converted to percentage of dose recovered (PDR) and curves are fit to the series of breath samples from each participant breath. From these breath curves, the time at which 50% of the final test dose is recovered (T_50_) will be considered as a primary outcome of interest. This choice is based on prior work that demonstrates that T_peak_ and T_50_ are associated with intestinal sucrase activity compared with other aspects of sucrose metabolism (eg, the fraction of the plasma bicarbonate excreted on the breath as opposed to excreted in urine or sequestered).^36^ Other summary measures of the ^13^C-SBT such as cumulative PDR by 90 min may also be influenced by misspecifications of CO_2_ production rate (VCO_2_), which is based on the participant’s estimated body surface area and sex.^52^ The DSIT is summarised as the % digestibility of specific amino acids from spirulina. The method of estimation is summarised in Devi *et al*.^21^ Weight-for-age Z-scores will be calculated based on WHO reference standards.^53^ Plasma concentrations LPSBP and iFABP and faecal concentrations of MPO and NEO will also be analysed.

For all statistical analysis, p values <0.05 will be considered statistically significant and intention-to-treat analyses will be applied. Both our primary and secondary outcomes will be analysed as change from baseline to endline. Because LR ratios are generally not normally distributed, we will use the non-parametric Wilcoxon rank-sum test to compare the supplementation and control groups within each site. For combined analyses we will use logistic regression models (outcome: LR ratio above vs below EE cut-off, exposure: intervention or control group). For all secondary outcomes, we will similarly use the non-parametric Wilcoxon rank-sum test to compare the supplementation and control groups within each site. For combined analyses we will use linear regression models or logistic regression models based on appropriate cut-off values, including a random effect to account for clustering by site.

### Patient and public involvement

Participants were not involved in the design and conduct of this research. However, the priority of the research question is based on the public health priorities of the participating sites, and the protocol for each site (planned recruitment and procedures) is based on each participating institution’s long history of conducting related research with similar participant groups. Local community representatives will be informed of the project prior to recruitment and results will be disseminated to local policymakers and community representatives in accordance with each site’s established strategies for community engagement.

## Ethics and dissemination

The study protocols have been approved by the institutional review boards relevant to each study site:

*India*: Institutional Ethical Committee (IEC), Ground Floor, St. John’s Medical College, St John’s National Academy of Health Sciences, Study number 198/2023 (Approval date: October 2024, India) email: sjmc.ierb@stjohns.in

*Ghana*: Ghana Health Service Ethics Review Committee, Research and Development Division Ghana Health Service, P.O. Box MB190, GA-050-3303, Study number GHS-ERC (Approval date: 4 October 2024) email: ethics.research@ghsmail.org

*Malawi*: Kamuzu University of Health Sciences Research and Ethics Committee (KUREC), Study Number P.02233992 (Approval date: 15 November 2023, Malawi) email: sec.rsc@kuhes.ac.mw

*Morocco*: Ethics Committee for Biomedical Research, Faculty of Medicine and Pharmacy, Rabat. Ref CERB 22/23 (Approval date: 13 May 2024, Morocco) email: fmp@um5.ac.ma

Philippines: Food and Nutrition Research Institute Institutional Ethics Review Committee (FIERC), Study number FIERC-2023-004 (Approval date: 28 June 2024, Philippines) email: fierc.fnri@gmail.com

*Zambia*: University of Zambia Biomedical Research Ethics Committee, Study number 5358–2024 (Approval date: 15 June 2024, Zambia). email: unzarec@unza.zm

The studies are also registered at CTRI (CTRI/2024/06/069187 in India); ClinicalTrials.gov (NCT06617130 in Malawi, NCT06676215 in the Philippines and NCT07256028 in Morocco); Pan African Clinical Trial Registry (pactr.samrc.ac.za) with identification number for the PACTR202311714091884 and the Ghana Food and Drug Authority in Ghana; Ongoing (Morocco); Ongoing (Zambia). Each site will generate locally specific recruitment materials and consent forms (online supplement 3). Written informed consent will be obtained from the legal guardians of all participants by trained members of each study team. We will publish and disseminate our results once the project is complete, with primary outcomes to be published together, in a single publication. Each site will communicate important protocol modifications to local regulators and to the overall study consortium.

## Discussion

Reducing the burden of suboptimal growth and development remains key to promoting child well-being worldwide. Our study aims to better understand whether amino acid supplementation can promote gut health among stunted children. While we are limited in our inability to examine long-term outcomes, such as improvements in linear growth associated with the intervention, improvements in indicators of gut function and macronutrient absorption (ie, intestinal absorptive capacity and protein digestibility) are important surrogate outcomes with biologically plausible and previously observed associations with linear growth.

A strength of our study design is the inclusion of children from diverse low- and middle-income country settings, which is key to understanding the impact of the intervention across different contexts where the aetiology of stunting may vary. In addition, our use of stable isotope-based tests to directly assess protein digestibility (using the DSIT) and carbohydrate absorption (using the ^13^C-SBT) provide direct measures of gut function, as opposed to indirect measures of intestinal inflammation or permeability.

## Supplementary material

10.1136/bmjopen-2025-105456 online supplemental file 1

10.1136/bmjopen-2025-105456 online supplemental file 2

10.1136/bmjopen-2025-105456 online supplemental file 3
