# Supplementary material for: Impact of indispensable amino acid supplementation on gut function in children at high risk of environmental enteropathy: protocol for an international coordinated group of randomised controlled trials
Source: BMJ Open. 2026 Apr 24;16(4):e105456. doi: 10.1136/bmjopen-2025-105456 (PMC13110547; doi:10.1136/bmjopen-2025-105456)
Supplement: online supplemental file 1 [file bmjopen-16-4-s001.pdf]

CONSENT FORM

1.1 STUDY TITLE: **The Efficacy of Amino Acid Supplementation in Treating Environmental Enteric Dysfunction Among Children at Risk of Malnutrition; A Randomized Controlled Trial - GHS-ERC 010/04/24**

PARTICIPANTS' STATEMENT

I acknowledge that I have read or have had the purpose and contents of the Participants' Information Sheet read and all questions satisfactorily explained to me in a language I understand (Akan, Ewe, Ga, Hausa, Dagbani). I fully understand the contents and any potential implications as well as my right to change my mind (i.e. withdraw from the research) even after I have signed this form.

I voluntarily agree to be part of this research.

Name of Participant..............................

Parents' or Guardian's Signature on behalf of the child. ....

OR Thumb Print of parent or guardian on behalf of the child ....

Date:......................................

INTERPRETERS' STATEMENT

I interpreted the purpose and contents of the Participants' Information Sheet to the afore named participant to the best of my ability in the (Akan, Ewe, Ga, Hausa, Dagbani) language to his proper understanding.

All questions, appropriate clarifications sort by the participant and answers were also duly interpreted to his/her satisfaction.

Name of Interpreter................................

Signature of Interpreter............................ OR Thumb Print ....

Date:...........................

Contact Details

....

This Is To Certify That This Study's Informed Consent Form Has Been Approved By The GHS ERC For The
Period 15/10/24 To 24/10/25
Sign.............. Date 22/10/24
Name Zelma Okine
GHS ERC Administrator
for

STATEMENT OF WITNESS

I was present when the purpose and contents of the Participant Information Sheet was read and explained satisfactorily to the participant in the language, he/she understood (Akan, Ewe, Ga, Hausa, Dagbani)

I confirm that he/she was given the opportunity to ask questions/seek clarifications and same were duly answered to his/her satisfaction before voluntarily agreeing to be part of the research.

Name:..............................

Signature................................ OR Thumb Print ....

Date:.................................

INVESTIGATOR STATEMENT AND SIGNATURE

I certify that the participant has been given ample time to read and learn about the study. All questions and clarifications raised by the participant have been addressed.

Researcher's name...

Signature ....

Date...

for

This Is To Certify That This Study's In[illegible]d Consent Form Has Been Approved By The GHS ERC For The
Period 15/10/24 To 14/10/25
Sign [illegible]
Name Zelma Da 22/10/24
GHS ERC Administrator

SUBJECT INFORMATION SHEET

To measure the protein digestibility and absorption by minimally invasive stable isotope-based methods and to assess the effect of amino acid based intervention on Environmental Enteric Dysfunction

The following information will be provided (in English or Kannada) to mothers/caretakers of infants of 18-36 months (<3 years) recruited to the study. In the case of illiterate parents/guardians the information will be read to them in the language of their choice.

Dear Parent,
We seek your permission to allow your infant to take part in a study entitled "**To measure the protein digestibility and absorption by minimally invasive stable isotope-based methods and to assess the effect of amino acid based intervention on Environmental Enteric Dysfunction.**"

Who is conducting this study?
This study is being conducted by researchers from the St. John's Research Institute, St. John's National Academy of Health Sciences, Sarjapur Road, Koramangala, Bangalore. This study has been approved by the Institution Ethics Committee of St. John's Medical College, Bangalore.

Why do we want to conduct this study?
Environmental Enteric Dysfunction (EED) refers to an inability to absorb nutrients in the stomach after food is being consumed. This problem is common in children living in low- and middle-income country settings with poor environment in terms of sanitation. EED is a poorly understood condition that may have far-reaching impacts on child health, development and strongly known to be associated with stunting (low height-for-age). Therefore, it is important to study its causes, and effects and possible preventive or curative approaches. Towards this, we aim to study the protein digestion and absorption in infants. Since, there is limited data on the impact of EED on the dietary protein in infants, we also aim to use an amino acid-based intervention (indispensable, based on the requirements) along with legume based meal for 28 days in 18-36 months old infants, to assess its role in treating EED, and to generate new data for protein metabolism and gut health.

What will you have to do?
Your infant (if >18 months to <36 months, stunted i.e., length-for-age (LAZ<-2) will be screened for EED and non-EED, their nutrient absorption and digestibility will be studied by the breath test and dual stable isotope technique respectively. For each participant, tests to be conducted at study enrolment will be spread over two days. Similarly, at the end of the study (day 28), endline tests will be spread over 2 days. The entire study will take about 1 month. Before starting at baseline and end at endline the following protocols will be conducted.

1. Breath test: after one baseline breath sample collection from your infant, sugar solution in water will be given to drink following which breath samples will be collected every 20 minutes for a total of 4 hours by using customized breath bags. You and your infant will have to stay in the division of nutrition for about 4 hours. Your child will be provided with water and/or milk over the first 90 mins of the study as needed.

2. For digestibility measurement on the experiment day, your infant will be fed legume based plant protein (mung bean) cooked in traditional home-based recipe (Khichdi). Baseline blood (2 mL), breath and urine (10 mL) will be collected, and breath samples will be collected every 20 mins over

360 mins (6 hours) throughout the study with additional two blood samples at plateau state (between 5th -6th hrs).

3. For L/R test- On study day 0, the lactulose-rhamnose test (L/R) will be conducted alongside the digestibility test. A single urine sample will be collected before the start of the study and the time noted. At the start of the study period (time=0), lactulose (1g) and rhamnose (0.2g) will be given to your infant with 100 mL water. All urine passed from 30 minutes up to 5hrs is collected and pooled and the total volume noted. Water or milk can be freely consumed throughout the study period.

4. Height & Weight- The first anthropometric measurement will be taken at the screening visit (study day 0), because your child's weight is needed to calculate the dosage for the breath test and dual stable isotope tests on days 1 and 2. The second anthropometric assessment will be made on study day 28 (after the intervention period).

The assessment will start within two weeks of enrollment to the study. Following the baseline study assessments, your infant will receive the standard complementary foods (In the control group), or standard complementary foods supplemented with amino acids (building blocks of proteins for the body) in the intervention group will be taken once daily by your infant for 28 days.

Your child's participation in the study
Your participation will be for 28 days from the recruitment till the end of the study. The study staff will guide and direct you throughout, until the completion of the study.

The decision to enroll in the study is voluntary and you will not lose any benefits if you choose not to participate in the study. You are free to withdraw your participation at any time during the study. You are free to refuse to answer any questions that you do not like or refuse certain specific procedures.

What is the "risk" to you?
a) There will be no risk to your infant or you. The stable isotope labelled proteins, sugars given are safe and non-radioactive as well as amino acid fed are perfectly safe and are needed for your infant for growth and will not cause any harm.
b) The pain of needle injections.
c) The discomfort of collecting breath, urine and feces which is unlikely.

Are there any benefits in participating in the study?
There is no direct benefit from participating in this study. It will help us understand digestibility of proteins, absorption of nutrients during stunting and will help us to know whether healthy changes in body occur in EED or non-EED infants after giving supplementation. However, there will be compensation for travel and other medical allowance if the participant is visiting the hospital only for research purposes.

Will my child's information be kept private?
The study team will keep the information provided confidential. All study documents will be stored safely with only the study team having access to them. Your personal details like name and address will not be used in any of the scientific reports or publications of the study.

How will we use the information?
The results we obtain from this study will help inform us about improving growth and nutritional status of stunted infants. The information will also be used for scientific publications while confidentiality is maintained.

Whom can you contact for any further information?
You understand that in the case of any problem you can contact Principal Investigator, Dr. Sarita Devi, Lecturer, Division of Nutrition, St. John's Research Institute (Tel. +91-80-49467000, Mobile No. 9986426938, sarita@sjri.res.in) or Co-Principal Investigator, Dr. Anura V Kurpad, Professor, Department of Physiology and Nutrition, St. John's Medical college (Mobile No. 9686512233, a.kurpad@sjri.res.in).

Also, for any clarification about your rights as a research subject, you may contact Dr. Jayanthi Savio, Member secretary, Institutional Ethical Committee, Ground Floor, St. John's Medical college, Sarjapur Road, Bangalore-560034, Ph: 080-49466346/48, Mail: sjmc.ierb@stjohns.in.

Thank you.

CONSENT FORM

To measure the protein digestibility and absorption by minimally invasive stable isotope-based methods and to assess the effect of amino acid based intervention on Environmental Enteric Dysfunction

My infant and I, voluntarily agree to participate in this study entitled **"To measure the protein digestibility and absorption by minimally invasive stable isotope-based methods and to assess the effect of amino acid based intervention on Environmental Enteric Dysfunction"** I confirm that I have understood the Information sheet for the above study and have had the opportunity to ask questions. The nature and demands involved in this study have been fully explained to me. I understand that me and my infant may withdraw from this study at any time for any reason and that I will not have to pay for the procedure. In the unlikely event of physical injury resulting from participation in this research, I understand that medical treatment will be available for my child from the St. John's Medical College and Hospital including first aid, emergency treatment and follow-up care as needed, and the cost and travel expenses will be borne by the Investigators. However, no compensation can be provided for medical care apart from the foregoing. I also received a copy of the information sheet to retain. I understand that the study will involve interviews to collect personal details, food intake of my infant and measurement of my infant height, weight, mid upper arm circumference and triceps skinfold. Feeding my infant, legume based plant protein (mung bean) cooked in traditional home-based recipe (Khichdi), or sugar or mixture of amino acids with multiple micronutrients based on his/her eligibility for the study. Monitoring my infant for daily compliance. Collection of blood, breath, urine, and fecal samples before and during the study.

I understand that the information I provide about me, and my infant will be used only for the purpose as described. They will not be used in any way that is harmful or discriminatory against me or my family. I also understand that by my participation in this study, I am not waiving any of my legal rights. I understand that in the case of any problem, I can contact Principal Investigator or Co-Principal Investigators of this study.

I give my permission to be enrolled in the study along with my child. **Child ID: _____________**

Name of the Respondent   Thumb Impression/Signature & Date

Name of the Interviewer (s)   Thumb Impression/Signature & Date

Name of Witness   Thumb Impression/Signature & Date

PARTICIPANT INFORMATION SHEETS AND CONSENT FORMS

Appendix 1a: Participant information sheet, English

Title: The Efficacy of Amino Acid Supplementation in Treating Environmental Enteric Dysfunction Among Children at Risk of Malnutrition

Purpose of the study

You are being asked to participate in a study that the Kamuzu University of Health Sciences is doing in Mangochi district. The purpose of the study is to assess the impact of essential amino acid nutrient supplementation on intestinal health among children at risk of stunting. We believe that the results of the study will help us to find better means of nutrient supplementation in improving intestinal health to prevent stunting. In our previous studies we have shown that children that are stunted have poor intestinal health and low levels of amino acids which are nutrients that help children grow well.

Procedures of the study

If you decide that your child should participate in the study, you will be requested to do come to the clinic on 5 separate days, three days at the beginning and 2 days towards the end of the study.

On the first day you come to the clinic you will spend 2-3 hours at the clinic during which time we will assess your child to see he or she can participate in the study. This assessment will include measuring the height and weight of the child, checking for anaemia and asking you some questions about yourself and your household. If your child is eligible to participate in the study and you are willing that the child participates, we provide you detailed information about what will happen during the study, and we will ask you to discuss with your family if you still want to participate after receiving the additional information. We will contact you on the evening of this day to check whether you are willing to participate or not.

On the second day after you have discussed and agreed with your family, we will send a driver to pick you from home and bring you to the clinic and ask you to provide consent to participate and this will be done by signing a form or thumb printing a form. When you have made the decision, you are welcome to bring a relative with you to further discuss the study before providing your informed consent. You will be asked to sign two forms and the project team will also sign on both consent forms. You will take one signed consent form while the other form will be kept by the project team. On this day we will ask you to choose an envelope that will tell your child’s identification number for the study and put him or her in one of two study groups. Participants in one study group will be receiving standard Likuni porridge mixed with the nutrient supplement containing amino acids while those in the other group will just receive Likuni porridge without any added supplements.

On the same day after you have given permission to participate in the study, we will collect a breath sample from your child using a mask and bag. Then we will give your child a solution of water and a special type of sugar to drink. We will continue collecting breath samples every 20 minutes for a total of 4 hours. During the four hours your child can only drink other water or breast milk during the first hour and 30 minutes, but after that they can eat other foods. During this day we will also ask you about the usual foods that you give to your child. This means if you come at 7am in the morning you will stay at the clinic until about 1pm. At the end of this day, we will also take you in our car and drop you home.

On day 3, we will come again to collect you from your home in the morning to come to the research office. We will ask you to bring a stool sample of the child. Once you are at the clinic, we will do the following:

1) We will ask that your child should not have eaten anything during the one hour before we start the clinic activities.
2) We will collect a urine sample using a collection bag. Then we will give your child a drink containing a special sugar that helps us measure how well the intestine is working.
3) We will insert a cannula in the arm of your child so that we can collect blood easily. We will collect 3ml of blood from the vein.
4) We will prepare Likuni porridge and share the porridge with you and the child. If child is the group receiving Likuni porridge with added amino acids, you will also receive a similar porridge. So that you can test what the child will be eating. We will put aside 9 mini portions (2 tablespoons) of the child porridge and give the first three portions at the start and a portion each at end of every hour until 5 hours. For children receiving the amino acid supplements, their porridge will be sprinkled and mixed with the amino acids every time before they eat the porridge.
5) At 5 hours, we will collect 3 blood samples of 3 mls each at 5 hours, 5.5 hours, and 6 hours.
6) We will also collect a urine sample at 5 hours.

After five hours we will take you home in our vehicle.

Between day 4 and day 30 we will provide you a weekly supply of Likuni porridge flour so that you can prepare porridge at home and give it to the child. If your child is in the group receiving Likuni porridge and amino acid supplements you will also receive amino acid which you will be trained to measure and add to the child's porridge. During this period our research assistant will come to your home every second day to observe the child feeding the porridge.

On day 32 and 33 we will come to collect you to come to the clinic and repeat the measurements we conducted on the second and third day.

Benefits

There are no direct benefits to the child for participating in the study. For each visit you come with your child to the clinic you will be compensated MK 15000 for your time and transport costs. You will be given MK1000 for each completed home visit. During the study period all your children will receive normal health care.

Risks

Drawing blood may cause some pain or discomfort to the child. It may also result in tenderness and bruising at the puncture site. However, these will be short-lived. Dosages of the nutrient supplement in the range used in this intervention are rarely associated with reported side effects. Apart from these, the study has no known risks although we cannot guarantee that any of the participants will not develop complications. Should those happen, we will help you access treatment within the government health system, but we are not providing any insurance for any potential harmful effects that may happen which we do not know already.
After we have completed our laboratory analyses, there will be some left-over breath, blood, urine, and stool samples from your child, and we ask your permission to keep those samples for future research. If we do any further analyses in the future from these samples, you would not be contacted again, but we would seek permission from the College of Medicine Research and Ethics Committee, COMREC

Cost

If you take part in this study, it will not cost you or any member of your family any money

Confidentiality

The records of this study will be kept securely at the Kamuzu University of Health Sciences and your child’s personal details will not be known by or released to anybody other than the project team. Unique identification number will be assigned to your child and all information including samples collected will be identified using this number and not the child’s name.

Right to refuse to participate or withdraw from the study.

You have the right to refuse your child takes part in the study. You may also change your mind about your child taking part in the study and withdraw your child´s participation in the study at any time. You do not need to give any reason for withdrawing your consent for your child to participate.

Questions

We will be happy to answer any questions you have about this study. If you have a project-related problem, you may contact the principal investigator (Prof. Kenneth Maleta, at 0888 232 202). If you have any questions concerning your child’s rights as a study participant, you may contact the Institutional Review Board Chairman at Kamuzu University of Health Sciences, Assoc. Prof Eric Umar (Cell: xxxxxxxxxx).

Appendix 1b: Participant information sheet, Chichewa

Mutu: Kafukufuku wopima mphamvu za michere ya mthupi pochiritsa matenda a m'matumbo mwa ana omwe ali pachiopsyezo cha kunyentchera/kupinimbira

Cholinga cha kafukufuku

Mukupemphedwa kuti mwana wanu atenge nawo mbali mukafukufuku yemwe sukulu yazaukachenjede ya Kamuzu University of Health Sciences ikuchititsa kuno ku Mangochi. Cholinga cha kafukufukuyi ndi kupima mphamvu zamichere ya mthupi yomwe imaperekedwa kwa ana pa matenda a m'matumbo mwa ana omwe ali pachiopsyezo cha kunyentchera/kupinimbira. Zotsatira zakafukufukuyi zizathandiza kupeza njira zabwino zokhwimisira matumbo ndicholinga chopewa kunyetchera/kupinimbira mwa ana. Kafukufuku wam'mbuyo yemwe tinachitapo anawonetsa kuti ana omwe ali wonyentchera/wopinimbira amakhala ndi matumbo osakhwima komanso michere yochepa yomwe imafunika kuti ana akule bwino.

Ndondomeko wa kafukufuku

Ngati mungavomereze kuti mwana wanu atenge nawo mbali mukafukufukuyu, muzapemphedwa kubwera kuchipatala masiku 5. Pamasiku 5 amenewa, masiku atatu muzabwera koyambirira kwakafukufuku ndipo masiku awiri enawo muzabwera kumapeto wakafukufuku.

Pa tsiku loyamba lomwe muzabwere kuchipatala muzakhala ma ola okwanira awiri kufikira atatu ndipo akafukufuku azamupima mwana wanu ngati ali woyenera kutenga nawo mbali mukafukufuku. Mwana wanu azayezedwa kutalika, sikelo, ngati ali ndi magazi okwanira komanso inu muzafunsidwa mafunso okhuza inuyo ndi kunyumba kwanu. Ngati mwana wanu ali woyenera kutenga nawo mbali mukafukufukuyi komanso inu mukuvomera kuti mwana wanu atero, muzafotokozeredwa ndondomeko yonse yakafukufukuyi mwatsatanetsatane ndipo muzapemphedwa kukafotokozerana ndi akubanja kwanu ngati mukuvomerabe kuti mwana wanu atenge nawo mbali mukafukufukuyi. Madzulo, akafukufuku azakuyimbirani foni kapena kubwera kunyumba kwanu ngati mulibe foni kuti azamve chiganizo chanu kuti mwana wanu atenge nawo mbali mukafukufuku kapena ayi.

Pa tsiku lachiwiri ngati inu ndi banja lanu muzagwirizane kuti mwana wanu atenge nawo mbali mukafukufuku, akafukufuku azakubweresani kuchipatala pa galimoto yawo ndi cholinga chakuti muzatsimikize kuvomera kwanu pakusaina kapena kudinda pazikalata za chilolezo. Mutha kuzabwera ndi wachibale kuti muzakambisane mwamvemve ndi akafukufuku musanapereke chilolezo chanu. Muzapemphedwa kupereka chilolezo chanu pakusaina kapena kudinda pa kalata ziwiri za chilolezo. Nawonso akafukufuku azasaina pa zikalatazi. Inu muzatenga kalata imodzi ndipo inayo izasungidwa mwachinsinsi ku ofesi ya akafukufuku. Muzapemphedwa kusankha invelopi imodzi yomwe izaonetse nambala yachinsinsi ya mwana wanu komanso ndondomeko yomwe iye azasatire mukafukufukuyi. Mukafukufukuyi ana azizalandira phala la Likuni

losakaniza ndi michere ya amino asidi ndipo ena azizalandira phala la Likuni lokha lopanda michere ya amino asidi.

Pa tsiku lomweli lomwe mwapereka chilolezo kuti mwana wanu atenge nawo mbali mukafufukufuku, tizayeza mpweya wa mwana wanu pogwiritsa ntchito masiki ndi bagi. Mwana wanu azapatsidwa madzi a shuga kuti amwe. Tizapitiriza kuyeza mpweya wa mwana wanu mphindi 20 zilizonse kwa ma ola 4. Mu ma ola 4 amenewa mwana wanu azaloredwa kumwa madzi ndi mkaka okha kwa ola ndi theka zoyambirira ndipo ma ola otstirawo azaloredwa kudya chakudya chilichonse. Pa tsiku lomweli muzafunsidwa za chakudya chomwe mumamupatsa mwana wanu nthawi ndi nthawi. Izi zikutanthauza kuti ngati muzafike 7 koloko mamawa kuchipatala muzakhala mpaka 1 koloko masana. Pakutha pa zonse akafukufuku azakakusiyani kunyumba kwanu pa galimoto.

Pa tsiku lachitatu akafukufuku azakutengani kunyumba kwanu mamawa kubwera nanu kuchipatala. Muzapemphedwa kubweretsa chimbuzi cha mwana wanu. Pa tsikuli akafukufuku azapanga izi:

1) Muzapemphedwa kuti mwana wanu asadye china chilichonse mmene mwafikira kuchipatala mpaka patatha ola limodzi.
2) Azatenga mkodzo wa mwana wanu pogwiritsa ntchito dayipa. Mwana wanu azapatsidwa chakumwa chothira shuga kuti amwe ndipo izi zimathandiza kuyeza mmene matumbo amwana akugwirira ntchito.
3) Azayika kanula padzanja la mwana ndicholinga chofuna kutenga magazi ake mosazuta. Azatenga magazi ochepa okwana mulingo wa tisipuni (3ml) kusempha wake
4) Muzapatsidwa phala la likuni inu ndi mwana wanu. Ngati mwana wanu ali mugulu lolandira phala la likuni lothiramo michere ya amino asidi ndiye kuti muzalandira phala lotero. Cholinga cha izi ndi kuti mulawe zomwe mwana wanu akupatsidwa kuti adye. Phala la mwana lizagawidwa m'magulu 9 ndipo gulu lirilonse lizakhala lokwana mulingo wa ma tebosipuni awiri. Mwanayo azadya magulu atatu aphala oyambirira pakamodzi ndipo magulu aphala enawo azizadyedwa pakutha pa ola lirilonse kufikira patatha ma ola 5. Ana omwe akulandira phala lothira michere ya amino asidi, gulu liri lonse la phala lawo lizizathiridwa michereyi asanadye.
5) Pakutha pa ma ola 5 tizatenga masampo atatu amagazi okwana mulingo wa tisipuni (3ml). Sampo yoyamba yamagazi izatengedwa pakutha pa ma ola 5, sampo yachiwiri izatengedwa pakutha pa ma ola 5 ndi theka ndipo sampo yachitatu izatengedwa pakutha pa ma ola 6.
6) Azatenga mikodzo kuchoka kwa mwana wanu pakutha kwa ma ola 5

Pakutha pa ma ola 5 akafukufuku azakakusiyani ku nyumba kwanu pa galimoto.

Pakati pa masiku 2 ndi 30 mwana wanu ali mukafukufuku, muzizapatsidwa ufa wa phala la Likuni lokwanira sabata imodzi kuti muzikaphika phala kunyumba loti mwana wanu ali mukafukufufku azidya. Ngati mwana wanu ali mugulu lolandira phala la likuni lothiramo michere ya amino asidi ndiye kuti muzalandiranso michere ya amino aside. Akafukufuku azakuphunzitsani kayezedwe ka mulingo wa michere ya amino asidi yomwe muzizathira m'phala la mwana. Munthawi imeneyi akafukufuku azizakuyenderani tsiku lachiwiri lirilonse kuti azizaona mmene mwanayo akudyera phala.

Pa tsiku la 32 ndi 33 akafukufuku azakutengani inu ndi mwana wanu kunyumba kwanu kubwera nanu kuchipatala kuti azapangenso ndondomeko zomwe zinachitika pa tsiku lachiwiri ndi lachitatu zomwe tafotokoza mumtundamu.

Phindu lotenga nawo mbali mukafukufuku

Palibe phindu lapaderadera kwa mwana wanu chifukwa chotenga nawo mbali mukafukufukuyi. Paulendo wina uli wonse mukubwera kuchipatala ndi mwana wanu muzapatsidwa ndalama zokwana MK15000 chifukwa cha nthawi yanu. Pamene mwayenderedwa kunyumba kwanu ndi akafukufuku muzapatsidwa ndalama zokwana MK1000 chifukwa cha nthawi yanu. Munthawi yonse yakafukufuku mwana wanu azalandira chisamaliro ngati ana ena onse kuchipatala cha boma.

Chiopsezo

Kafukufukuyi alibe chiopsezo kwa mwanu wanu. Nthawi zina munthu amatha kumva kuwawa pang’ono akamatengedwa magazi koma kuwawako sikukhala nthawi yayitali. Mulingo wa michere yomwe ikuperekedwa ilibe chiopsezo muthupi kwenikweni. Komabe patakhala kuli pali vuto lam’thupi pamene mwana wanu akutenga nawo mbali mukafukufukuyi tizakuthandizani kupeza chithandizo choyenera kuchipatala cha boma. Sitizapereka chithandizo china chapaderadera kuposa icho muzapatsidwe ku chipatala cha boma.
Pamene tamaliza kuunika zonse mukafukufukuyi, ngati pazasale masampo ochokera kwa mwana wanu a mpweya, magazi, mikodzo ndi chimbuzi tikupempha chilolezo chanu pano kuti tizathe kusunga masampowo ndikuzawagwiritsa ntchito mukafukufuku wina wamtsogolo. Choncho sitizabweranso kwa inu kuti tipemphe chilolezo koma tizakapempha chilolezo ku bungwe loyendetsa kafukufukuku la COMREC kusukulu yaukachenjede ya Kamuzu University of Health Sciences.

Kuononga ndalama zanu

Inu, mwanu wanu kapena wina aliyense wakubanja kwanu sazawononga ndalama zake chifukwa choti mwana wanu akutenga nawo mukafukufukuyi

Kusunga chinsinsi

Uthenga wonse komanso masampo otoleredwa mukafukufukuyi zizasungidwa mwachinsinsi kusukulu yaukachenjedwe ya Kamuzu University of Health Sciences ndipo sizizaperekedwa kwa wina aliyense kupatula iwo akuyendesa kafukufukuyi. Tizagwiritsa ntchito nambala ya chinsinsi pa uthenga ndi masampo ake onse ndipo sitizagwiritsa ntchito dzina lake

Ufulu wanu kusiya kapena kukana kutenga nawo mbali mukafukufukuyi

Dziwani kuti muli ndi ufulu kukana kuti mwana wanu asatenge nawo mbali mukafukufukuyi. Mwana wanu atha kusiya kutenga nawo mbali mukafukufukuyi nthawi ina ili yonse yomwe inu mwafuna ndipo simuli okakamizidwa kupereka chifukwa chomwe mwanayo akusiyira.

Mafunso

Khalani omasuka kufunsa mafunso ena ali onse muli nawo okhuza kafukufukuyi ndipo tikuyankhani. Ngati muli ndi mafunso ena owonjezera kapena ngati muli ndi vuto lokhuza kafukufukuyi mutha kuyimbira otsogolera kafukufukuyi Prof. Kenneth Maleta pa foni nambala iyi 0888 232 202. Ngati muli ndi mafunso okhuza ufulu wa mwana wanu kutenga nawo mbali mukafukufukuyi mutha kuyimbira mkulu woyang'anira kafukufuku ku sukulu yaukachenjede ya Kamuzu University of Health Sciences, Assoc. Prof Eric Umar pa foni nambala iyi xxxxxxxxxx.

Appendix 1c: Participant information sheet, Yao

Mtwe: Kawungunya jwakupima machili ga njete sya mchiru pakuposya mawulwere ga mmatumbo mwa wanache wali pachogoyo cha usemwe.

Chakulinga cha kawungunyaju

Tukwawenda kuti mwanache jwawo ajigale nawo mblai mukawungunya jwa wa sukulu ja Kamuzu University of Health Sciences akutendesya akuno kumangochi. Chakulinga chakawungunyaju ni kupima machili ga njete sya mchiru jajikusaperechedwa kwa wanache pamaulwere gammatumbo mwa wanache wali pachogoyo chakutenda usemweYakuyichisya yakawungunyaju ichikamuchisya kupata matala gambone gakomangasya matumbo nichakulinga cha kuteteya usemwe wa wanache. Kawungunya jwamunyuma jutwatesile jwalosisye kuti wanache wali wa usemwe akusawaga ni matumbo gangakomala nambo soni njete jamnono jajikusasosegwa kuti wanache akule chenene.

Mlundunde wa kawungunyaju

Naga ajitichisye kuti mwanache jwawo ajigale nawo mbali mukawungunyaju, tuchawenda kwika kuchipatala masiku nsano. Pamasiku 5 gerega, masiku gatatu tachiyika pakutanda pakawungunyaju, nambo soni masiku gawiri ganego tachiyika kumapeto kwakawungunyaju.

Pa lisiku lyandanda litachiika kuchipatala tachitama ma awala gakwana gawiri mpakana gatatu soni wakafukufuku tachimpima mwanache jwawo naga ali jwakwenera kujigala nawo mbali mukawungunyaju. Mwanache jwawo tachilinjidwa ulewu, usito, naga jwana myasi jakwanira, nambo soni wawo tachiwusidwa mawusyo gakwayana ni wawojo nambo soni yakunyumba.Naga mwanache jwawo ali jwakwenera kujigala nawo mbali mukawungunyaju nambo soni wawo naga akundire kuti mwanache jwawo ajinjire, tachilongosololeredwa mlundunde wosope wa kawungunyaju chilichose soni tachawenda kuti akalongosolere wakunyumba kwawo, naga ali wakunda kwinjira nawo kawungunyaju.Ligulo wakawungunya tachajimbira foni kapena kwika panyumba pawonaga ali wangali foni kuti akapikane nganisyo syawo sya kuti mwanache jwawo tajinjire nawo kawungunyaju kapena iyayi.

Pa lisiku lyawili naga wawo ni wakunyumba chakamulane kuti mwanache jwawo atenge nawo mbali mukawungunyaju, wakawungunya tachiika nawo kuchipatala pagalimoto jawo ni chakulinga kuti takasimichisye kunda kwawo pakusaina kapena kudinda paikalata yachikundisyo. Tachikomboleka kwika ni wachibale kuti takamulane mwakupikanika ni wa kawungunya nkinakunde kwinjira nawo. Tachiwendedwa kupereka chikundisyo chawo pakusainila kapena kudinda paikalata iwili ya chikundisyo. Nombe nawo wakaungunya tachisainira ikalatayi. Wawo tachijigala chikalata chimo nambo soni chinecho chichisunjidwa mwachinsisi ku ofesi jakafukufuku. Tachiwendedwa kusagula inivelopi jimo jajichilosya nambala jachinsisi ja mwanache jwawo nambo soni ndondomeko jitachikuya mwanache jwawo mukawungunyaju.

Mukawungunyaju wanache tachipocheraga likoko lya likuni lyewanganye ni njete sya amino aside nambo wane tachiwaga ali nkupochera likokolya likuni lyangali njete sya amino asidi.
Pa lisiku liliri lyapereche chikundisyo kuti mwanache jwawo ajigale nawo mbali nkawungunyaju, tuchilinga mpweya wamwanache, pakamulichisya masengo masiki ni ka nsaku. Mwanache jwawo tachipochera mesi ga shuga kuti amwe. Tuchiwa tuli nkupitilira kulinga mpweya wamwanache pa mbindi 20 silisyose kwa ma awala 4. Mu ma awala 4 gerega mwanache jwawo tachikundisidwa kumwa mesi ni nkakape pa awalajimo ni hafu jandanda nambo soni ma awala gakuichisyago tachikundisidwa kulya chine chilichose. Pa lisiku liliri tachiwusidwa ya yakulya yakusampaga mwanache jwawo ndawi ni katema. Ayi igopolera kuti naga tayiche 7 koloko kundawi kuchipatala nikuti tachitama mpaka 1 koloko ja musi. Pakumala payosope wakafukufuku tachijakwaleka kunyumba kwawo pagalimoto.

Pa lisiku lyatatu wakakawungunya tachajigala kunyumba kwawo kundawi kwala nawo kuchipatala. Tachiwendedwa kwika nacho chimbusi cha mwanache. Palisikuli wakakawungunya tachitenda ayi:

1) Tachiwendedwa kuti mwanache jwawo akasalya chine chilichose chichire kuchipatala mpaka jimale awala jimo.
2) Tachijigala makweso ga mwanache jwawo pakamulichisya masengo pampa. Mwanache jwawo tachimpa chakumwa chetaje shuga kuti amwe, yereyi isakamuchisya kuti alinje mwagakamulira masengo matumbo gamwanache.
3) Tachiika kanyula pankono pamwanache kuti pawe pakujigalira myasi jamwanache mwangasausya. Tachijigala myasi jamnono jakwana ka supuni ka tiyi kamwana (3ml) pantasi wakwe.
4) Tachapa likoko lya likuni wawo ni mwanache jwawo. Naga mwanache jwawo ali mu gulu jakupochera likoko lyana njete sya amino aside nikuti nilitachipohera. Chakulinga nichakuti nombe wawo apasye yakupochera mwanache jwawo kuti alye. Likoko lya mwanache lichigawidwa mawupande 9 nambo soni upande uliwose uchiwaga wakwana ma supuni gekulungwa gawiri. Mwanachejo tachilya ma upande gatatu galikoko ndawi jimo nambo soni ma upande ganego tachilyaga pakumala pa awala jimo mpaka gakwane ma awala 5. Wanache wakupochera likoko lyetaje njete sya amino aside, upande uliwose wa likoko tiwuchitajidwaga njetesi nkinalye.
5) Pakumala pa ma awala 5 tuchijigala masampo gatatu gamyasi gakwana mlingo wa supuni ja tiyi jamwana (3ml). Sampo jandanda ja myasi jichijigalidwa pakumala ma awala 5, sampo jawiri jichijigalidwa pakumala pa ma awala 5 ni hafu, nambo soni sampo jatatu juchijigalidwa pakumala pa ma awala 6.
6) Tachijigala makweso kutyochera kwa mwanache jwawo pakumala pa ma awala nsano (5)

Pakumala pa ma awala nsano (5) wakawungunya tachijakwaleka ku nyumba kwawo pagalimoto.

Pakatikati pa masiku gawiri mpaka 30 mwanache jwawo ali mukawungunya, tachipocheraga utandi wa likoko kya kwana chijuma chimo kuti akaterecheje kunyumba kuti mwanache jwawo jwali mukawungunyaju alyeje. Naga mwanache jwawo tawe mgulu jakupochera utandi walikoko lyetaje njete sya amino aside nikuti tachipocheraga soni njetesyo. Wa kafukufuku tachaajiganya kalinje kakwe ka mlingo wa njete sya amino aside jakuti ataajeje mwikoko lyakulya mwanacheMundawi jereji wakawungunya tachiwaga alinkwajendera lisiku lyawiri lililyose kuti alore mwakulira likoko mwanache jwawo.

Palisiku lya 32 ni 33 wakawungunya tachajigala wawo ni mwanache jwawo kumangwawo kwika nawo kuchipatala kuti tatende mlundunde wine wa mpera uwatendeche palisiku lya wiri ni lyatatu yatulongwesere kala munyumamu.

Pindu jakujigala nawo mbali mu kawungunyaju

Pangali pindu jine jilijose japadera kwa mwanache jwawo jichachipata ligongo lya kujigala nawo mbali mukawungunyaju. Paulendo wine uliwose wutachiika kuchipatala ni mwanache jwawo tachipochera mbiya syakwana MK15000 kwayamika ligongo lya ndawi jawo. Naga wajendere kunyumba tachipochera mbiya syakwana MK1000 ligongo lya ndawi jawo. Mundawi syosope mwanache jwawo tachipochera chisamaliro chosope chakuchipatala mwangalekangana ni wanache wane pachipatala chaboma.

Chogoyo

Kawungunyaju jwangali chogoyo chilichose kwa mwanache jwawo. Katema kane mundu akusakombolekaga kupikana kupweteka panandi alinkujigalidwa myasi nambo kupwetekako kwangajigala ndawi jerewu. Mlingo wa njete jajikuperechedwa jangali chogoyo kwenikweni kuchilu. Nambope naga pali pana vuto janchilu pandawi jakujigala nawo mbali nkawungunyaju tuchakamuchisya kupata chikamuchisyo chakwenera kuchipatala chaboma. Ngasitupereka chikamuchisyo chine chapambali kupatulachichachipocherakuchipatala.
Patuchimalisya kumulika yosope mukawungunyaju, naga tipasigale masampo gakutyochera mwa mwanache jwawo ga mpweya, myasi, makweso ndi chimbusi tukwawenda chikundisyo chawo apano kuti titusunje masampogo nikujagakamulikchisya masengo mukawungunya jwine jwam'bujo. Nikuti, ngasituika soni kwa wawo kukuwenda chikundisyo nambo tuchijakuwenda ku likuga lya kwendesya achikafukufuku lya COMREC ku sukulu ja Kamuzu University of Health Sciences.

Konanga mbiya syawo

Wawojo, mwanache jwawo kapena jwalijose jwapamlango pawo ngisajonanga mbiya syawo paligongo lyakuti mwanache jwawo akujigala nawo mbali mukawungunyaju.

Kusunga Chinsisi

Mawutenga gosope ni masampo gichalokotanye mukawungunyaju ichisunjidwa mwachinsisi ku sukulu ja Kamuzu University of Health Sciences soni ngasiiperechedwa kwa jwine julijose nambo wakwendesya kawungunyape. Tuchikamulichisya masengo nambala ja chinsisi pa ngani ni masampo gawo ngasitukamulichisya masengo lina lyawo kapena lyamwanache.

Ukoto wawo kuleka kapena kana kujigala nawo mbali nkawungunyaju

Amanyilire kuti akwete ukoto kana kuti mwanache jwawo akajigala nawo mbali mukawungunyaju. Mwanache jwawo mpaka akomboleche kuleka kujigala nawo mbali nkawungunyaju ndawi jine jilijose jampaka asose soni nginawa wakakamisidwa kupereka ligongo lyakulechera mwanache jwawo.

Iwusyo

Atame wagopoka kuwusya mawusyo gane galigose gakwete gakafukufukuju chitwajanje. Naga akwete mawusyo gane gakonjechesya kapena naga akwete vuto jakuyana ni kawungunyaju mpaka akomboleche kwimbira wakulongolera kawungunyaju Prof. Kenneth Maleta pa nambala ja foni aji 0888232 202.Naga akwetemawusyo gakuyana ni ukoto wa mwanache jwawo kumbali jakujigala nawo mbali mukawungunyaju mpaka akomboleche kwimbira wakulungwa wakulolera achikawungunya wosope ku sukulu ja Kamuzu University of Health Sciences, Assoc. Prof Eric Umar pa nambala ja foni aji: xxxxxxxxxx.

Appendix 2a: Consent form, English

Title: The Efficacy of Amino Acid Supplementation in Treating Environmental Enteric Dysfunction Among Children at Risk of Malnutrition

I am going to read out statements to show if you agree that your child takes part in the study. I will record 'yes' against the statement if you ***agree*** with it and a 'no' if you ***do not agree***. If you answer yes to all the statements, I will ask you to confirm that you agree that your child takes part in the study by either signing or thumb printing on two consent forms. I will also sign the consent forms. You will collect one signed consent form while the other will be kept in the project team's secure office.

1. The project team has explained to me about the study, and I was a given a chance to ask questions where I did not understand Yes No
2. I agree that my child takes part in this study and will follow all the study procedures. Yes No.
3. I agree that biological samples of blood, breath, urine and stool be collected from my child during the study visits. Yes No
4. I agree that my child will consume the study meal as indicated by the project team. Yes No
5. I agree that my child be visited at home for observed feeding. Yes No
6. I agree that left-over biological samples collected from my child can be used for future research related to the current one and I will not be contacted again for permission. Yes No
7. I understand that all information and samples collected from my child will be kept securely and no name will be revealed on any of the materials. Yes No
8. I understand that I have the right to withdraw my child from the study at any time. Yes No

Name of participant ____

____________________	________________________	_ _/ _ _ _/_ _
Name of parent/care taker	Signature/thumbprint of parent/caretaker	Date
____________________	________________________	_ _/ _ _ _/_ _
Name of field worker/research nurse	Signature of field worker/research nurse	Date

Field worker/research nurse code ________________________________

Appendix 2b: Consent form, Chichewa

Mutu: Kafukufuku wopima mphamvu za michere ya mthupi pochiritsa matenda a m'matumbo mwa ana omwe ali pachiopsyezo cha kunyentchera

Ndikuwerengerani mfundo zakafukufukuyi zosonyeza kuvomera kwanu kuti mwana wanu atenge nawo mbali mukafukufukuyi. Ngati ***mwagwirizana*** nayo mfundo ine ndilemba eya pa kalata ya chilolezoyi kusonyeza kuvomera kwanu. Ngati ***simunagwirizane*** nayo mfundo ine ndilemba ayi pa kalata ya chilolezoyi kusonyeza kukana kwanu. Ngati mungagwirizane ndi mfundo zonse ndikupemphani kuti musaine kapena kudinda pa kalata ziwiri zachilolezo. Inenso ndisaina kalatazi. Inu mutenga kalata imodzi yosainidwa ndipo inayo izasungidwa motetezedwa ku ofesi ya akafukufuku

1. Akafukufuku andifotokozera zakafukufuku ndipo andipasa mwayi ofunsa mafunso. Eya Ayi
2. Ndikuvomera kuti mwana wanga atenge mbali mukafukufukuyi ndipo azatsatira ndondomeko zonse zakafukufukuyi. Eya Ayi
3. Ndikuvomera kuti mwana wanga azatengedwe masampo a mpweya, magazi, chimbuzi komanso mikodzo nthawi yakafukufukuyi. Eya Ayi
4. Ndikuvomera kuti mwana wanga azadya phala lomwe akafukufuku azizapereka. Eya Ayi
5. Ndikuvomera kuti mwana wanga azizayenderedwa kunyumba ndi akafukufuku kuzaona madyedwe ake a phala lakafukufuku. Eya Ayi
6. Ndikuvomera kuti masampo otsala otengedwa kwa mwana wanga azagwiritsidwe ntchito mukafukufuku wamtsogolo ogwirizana ndi kafukufuku wapanoyi opanda akafukufuku kuzapemphanso chilolezo kwaine. Eya Ayi
7. Ndamvetsa kuti uthenga ndi masampo ochokera kwa mwana wanga zizasungidwa motetezedwa ndipo akafukufuku sazayika dzina la mwana wanga pa zinthuzi. Eya Ayi
8. Ndamvetsa kuti ndili ndi ufulu womuchotsa mwana wanga mukafukufuku nthawi ina ili yonse. Eya Ayi

Dzina la mwana wotenga nawo mbali mukafukufuku ________________________________

______________________	__________________________	_ _/ _ _ _/_ _
Dzina la kholo/osunga mwana	Sayini/chidindo cha kholo/osunga mwana	Tsiku
______________________	__________________________	_ _/ _ _ _/_ _
Dzina la wakafukufuku	Sayini ya wakafukufuku	Tsiku

Code ya wakafukufuku _____________________________________

Appendix 2c: Consent form, Yao

Mtwe: Kaungunya jwakupima machili ga njete sya mchiru pakuposya ilwere ya mmatumbo mwa wanache wali pachogoyo chakunyentchera kapena usemwe.

Tinawalanjire mfundo sya kaungunyaju syakulosya kwitika kwawo kuti mwanache jwawo ajigale nawo mbali mukawungunyaju. Naga akamulane najo mfundo une tilembe elo pa chikalata cha chikundisyochi kulosya kwitika kwawo. Naga ***nginakamulana*** najo mfundo une tilembe iyayi pachikalata cha chikundisyochi kulosya kana kwawo. Naga ***takamulane*** ni mfundo syosope tinawende kuti asainile kapena kudinda pa ikalata iwiri yachikundisyoyi. Une soni tisainile ikalatayi. Wawo tajigale chikalata chimo chesainidwe soni jinejo jichisunjidwa ku ofesi ja kawungunya.

1. Wakawungunya alongosolere yakawungunyaju soni ambere upile wakuwusya mawusyo. Elo Iyayi
2. Ngwitichisya kuti mwanache jwangu ajigale nawo bali nkawungunyaju nambo soni tachikuya ndondomeko syosope syankawungunyaju. Elo Iyayi
3. Ngwitichisya kuti mwanache jwangu tajigalidwe ma sampo ga mpweya, myasi, chimbusi nambo soni makwesomu ndawi jakawungunya. Elo Iyayi
4. Ngwitichisya kuti mwanache jwangu tachilya likoko litachiperekaga wakawungunya. Elo Iyayi
5. Ngwitichisya kuti wakawungunya tamjendereje mwanache jwangu kunyumba kwisa kulora kalye ka likoko lya nkawungunya. Elo Iyayi
6. Ngwitichisya kuti masampo gakusigala gakujigalidwa kutyochera kwa mwanache jwangu gachikalichisidwa masengo mu achikawungunya wane wa m'bujo wakulandana ni kaungunya jwa leroju pangawendasoni kwawune. Elo Iyayi
7. Mbikanichisye kuti utenga nima sampo gakutyochera kwa mwanache jwangu ichisunjidwa mwakutetesedwa soni wakafukufuku ngisawika lina painduyi Elo Iyayi
8. Mbikanichisye kuti ngwete ukoto wakuntyosya mwanache jwangu nkawungunyaju ndawi jine jilijose. Elo Iyayi

Lina lya mwanache jwakujigala nawo mbali mukawungunyaju ______________________

________________________	___________________________	_ _/_ _ _/_ _
Lina lya nangolo	Saini/chidindo ja /wakusunga mwanache	Lisiku
________________________	___________________________	_ _/_ _ _/_ _
Lina lya wa kawungunya	Kusainira kwa wakawungunya	Lisiku

Code ja kawungunya ____________________

LETTRE D'INFORMATION

Utilisation des isotopes stables pour l'évaluation de la biodisponibilité des acides aminés indispensables chez des enfants présentant des entéropathies

Il s'agit d'une étude interventionnelle qui porte sur l'utilisation de techniques non ou peu invasives pour l'évaluation de l'assimilation des macromolécules et la biodisponibilité des acides aminés indispensables chez des enfants présentant des entéropathies. Cette étude est réalisée dans le cadre d'une collaboration entre l'Unité Mixte de recherche Nutrition et alimentation (CNESTEN – Université Ibn Tofail) et le Département d'Hépatologie Pédiatrique, Gastro-entérologie et Nutrition-P III, Hôpital d'Enfants, CHU Ibn Sina, Rabat.

L'objectif général : Evaluer l'absorption des nutriments et la biodisponibilité des acides aminés indispensables dans le cadre de l'EED et d'évaluer l'impact de certaines interventions (supplémentation en acides aminés / traitement médical) sur l'état nutritionnel de ces enfants.

Les objectifs spécifiques visent à:

1. Développer des approches isotopiques stables pour la surveillance de l'EED
2. Évaluer l'absorption des nutriments dans le contexte de l'EED
3. Quantifier les acides aminés indispensables mis à la disposition de l'organisme pour surmonter les troubles liés à l'EED.
4. Évaluer l'impact de certaines interventions pour réduire l'effet de l'EED
5. Evaluer la diversité microbienne chez els enfant ayant un EED et son évolution après le traitement

Est-ce que nous sommes obligés de participer à cette étude ?

Votre enfant n'est nullement obligé de participer à cette étude et que cette décision vous appartient entièrement. Toutefois, si vous souhaitez que votre enfant participe, veuillez lire et signer ce formulaire, un exemplaire signé et daté vous sera remis pour avoir toutes les informations concernant l'étude ainsi que les noms et les coordonnées des responsables de la recherche.
Vous pouvez interrompre la participation de votre enfant à tout moment sans avoir à vous justifier ni encourir aucune responsabilité, et votre décision n'affectera pas le traitement ou les rapports avec les médecins et l'équipe médicale.

Si nous acceptons de participer comment sera le déroulement de l'étude ?

Un médecin va vous faire un examen clinique et va prendre les mesures du poids et de la taille. L'équipe médicale, formée du médecin porteur du projet, du chercheur également porteur du projet et d'un (ou une) infirmier(e) spécialisé(e), vous donneront les repas contenant els traceurs. Elle veillera à la mise en place d'une voie veineuse périphérique, comme garde veine, ce qui permettra d'éviter de piquer plusieurs fois. Les prélèvements seront réalisés par du matériel à usage unique, tout en respectant les procédures d'usage en matière d'accidents et d'expositions au sang. Cette équipe se chargera aussi de faire les prélèvements d'air et d'urine.

Un membre de l'équipe se chargera de recueillir les informations nécessaires pour compléter les questionnaires individuelles et de fréquence alimentaire.

<u>Quels sont les risques et les dangers qui peuvent résulter de cette étude ?</u>

Il n'y'a aucun risque pour vous de participer à cette étude.

<u>La confidentialité de toutes les informations sera-t-elle préservée ?</u>

Toutes les informations concernant l'identité, le dossier médical et les résultats obtenus dans le cadre de cette étude seront traités de façon confidentielle.
Les données personnelles et médicales feront partie d'une base de données électronique traitée au laboratoire de l'Unité Mixte de Recherche en Nutrition et Alimentation.

<u>Comment seront publiés les résultats de cette étude ?</u>

Les résultats de cette étude pourront être publiés dans une revue scientifique. L'identité du participant n'apparaîtra dans aucun rapport ou publication et toute information sera traitée de façon confidentielle.

<u>Financement et assurance</u>

Toutes les visites et les consultations qui se dérouleront au cours de cette étude seront gratuites.

<u>Comité d'éthique</u>

Le Comité d'Ethique pour la Recherche Biomédicale (CERB) de la Faculté de Médecine et de Pharmacie de Rabat nous a accordé son approbation pour cette étude suite à la réunion en date du ….

<u>Qui contacter pour obtenir d'autres informations supplémentaires ?</u>

Pr. Toufik MESKINI **Tel. 06.61.41.22.41**
Mr. Mohammed EL MZIBRI **Tel. 06.61.47.91.24**

Nous vous remercions pour vos collaborations

FORMULAIRE DE CONSENTEMENT

Je, soussigné(e) . . . .

Parent / tuteur de l'enfant ………………………………………………………….

Certifie, avoir été informé(e) de toutes les informations relatives au projet intitulé « **Utilisation des isotopes stables pour l'évaluation de la biodisponibilité des acides aminés indispensables chez des enfants présentant des entéropathies**».

J'ai été sensibilisé(e) sur les objectifs exclusivement scientifiques de cette étude, et sur les résultats attendus.

Les données d'état-civil et de caractère médical qui me concernent et/ou concernent mon enfant, resteront **strictement confidentielles et protégées par le secret médical.**

J'accepte sous ces conditions de participer à cette étude, tout en gardant la faculté de me retirer de l'étude à tout moment.

Lu et approuvé.

Nom et Prénom : ____________________

Carte d'Identité Nationale : ____________________

Date / / Signature ____________________

INFORMED CONSENT FORM

PROJECT TITLE: **The Efficacy of Amino Acid Supplementation on Gut Function and Nutritional Status in Filipino Children Below Three Years Old**

NAME OF ORGANIZATION: **Department of Science and Technology- Food and Nutrition Research Institute**

PROJECT LEADER: **Amster Fei P. Baquiran**

You and your child are invited to join this study. This form has many parts for you to:

1. Know the procedures and criteria for selecting a participant
2. Know the procedures a qualified participant and his/her mother are expected to do
3. Affix your signature (or thumbmark, if illiterate) at the end if you wish to join in the study and comply with the requirements

PART I: INFORMATION SHEET

In summary, this study is a research study that involves the following:

1. Collection of information about you and your child (personal, demographic, anthropometric, food intake)
2. Collection of biological samples (breath, urine, stool, blood and saliva) from your child
3. Oral administration of solution or meal that contain sugars and/or stable isotope tracers for your child
4. Going to FNRI (you and your child) and house visits by study staff
5. Daily consumption of complementary food with or without supplement for 28 days

Details are as follows.

BACKGROUND ABOUT THE STUDY

The DOST-FNRI, together with 7 other countries around the world, is conducting a research study that needs the participation of children 18-36 months old and with stunting. Stunting is when a child has a low height for their age, and about 149 million or 22 percent of the world's children population around the world are stunted based on 2018 data of World Health Organization. It is being linked to a condition of small intestine called Environmental Enteric Dysfunction (EED). Children with EED have unusual features in their intestine which may cause poor nutrient intake, bacterial translocation, and inflammation leading to poor physical development. This study would help us understand EED and whether taking amino acid supplements would bring positive changes in a child's intestinal health and nutritional status.

PURPOSE OF THE STUDY

This study will look into the effects of amino acid supplementation on EED among children with stunting. There are different tests to be done to:

- Find out how much change in gut permeability or absorption using dual-sugar test after a 28-day supplementation
- Find out how much change, if any, in gut digestive capacity and plasma protein absorption index using stable isotope techniques
- Learn if supplementation cause changes in the levels of inflammatory markers
- Learn if supplementation changes the composition of gut microbiota
- Find out how much change, if any, in anthropometry (weight, height, weight-for-height z-score (WHZ), length/height for age (LAZ/HAZ)) and body composition (total body water, fat mass, fat-free mass, %fat) of the child after 6 months

PARTICIPANT SELECTION

Taguig and Paranaque are the chosen recruitment sites as they are close to FNRI where some procedures will be done. We will need 60 children as participants. To take part in this study, you (as the parent, caregiver, or guardian of the child) must be willing to give written, informed consent. Your child **must**:

- Be 18 to 36 months old (of either sex)
- Have stunting (LAZ/HAZ <-2)

Also, your child **must not**:

- Be wasted (WHZ <-2)

- Be overweight (WHZ >2)
- Have had diarrhea (by self-report) in the past month
- Have sibling or twin of another participating study child (2 children of different families in the same household are both eligible)
- Have taken antibiotics in the past month
- Have food allergies/intolerance
- Have a serious illness within the last three (3) months or have any underlying condition which may cause burden to your child to complete the process or would interfere in the analysis of study results

VOLUNTARY PARTICIPATION AND WITHDRAWAL

You and your child's participation in this study is voluntary. On every visit and test, you will be asked whether you would still want to participate in the study and will only proceed if given the consent. You may ask questions anytime if you need clarification and our study staff will be there to answer your concerns.

You have also the right to withdraw from the study at any time for any reason without consequence or penalty. You are free to choose to continue or withdraw from the intervention and/or from the study. In case the reason for discontinuation is unacceptable adverse events, one of our study staff will call you to follow up until the event is resolved.

Upon withdrawal/termination, any information and sample collected will be kept and processed as described in the protocol. But if you wish not to include your child's data in the analysis and reporting, we will remove your information from the records.

PROCEDURES

This part lists what will happen during the study and what you and your child are expected to comply with. A pediatrician will be joining us during the screening and data collection to attend to any medical concerns related to the study.

Screening

If you and your child agree to participate, you will sign this consent form. Then we will ask you some questions about your child, and your child will have some tests done to make sure he/she qualifies and it is safe for him/her to be in this study.

1. Collection of information about your child
2. Anthropometric measurement (height and weight)
3. Doctor's check-up (medical history and physical exam)
4. Collection of blood samples for screening (CRP + Hb)

The screening will last about an hour. Snacks will be provided.

If your child passed all the criteria, we will assign a unique study code to your child and we will randomly assign him/her into a group - intervention or control, which determines what food will be given to him/her. Both groups will undergo the same tests which are discussed in the following sections. We will give you a calendar of activities as your guide to what you and your child will do. We will also give you a food diary where you will list down the foods and beverages consumed by your child on certain schedules. A registered nutritionist-dietitian will interview you to validate the entries in the food diary.

Study Visit 1: Dual Sugar Test (L/R test), Deuterium Dilution Technique (DDT) and Sucrose Breath Test (SBT)

If your child qualifies, you and your child will be asked to visit our laboratory at FNRI, Bicutan, Taguig. Your child needs to be in a fasting state and no food, drinks or breastmilk is allowed 1 hour before the test. We will be giving your child a dose of sugar, heavy water and labelled sucrose. We will collect breath and saliva samples both before and after consuming the dose. Urine sample will be collected starting 30 minutes after your child consume the sugar dose and pooled for a total duration of 5 hours.

1. <u>Baseline breath collection.</u>

   Your child will be asked to blow in a clean plastic bag through a mouthpiece. The air in the bag will then be transferred in a clean vial. Two baseline samples will be collected at the start.

2. <u>Baseline saliva collection.</u>

A clean cotton swab (stick with cotton at the tip) will be used to collect saliva samples. A trained staff will swab the cotton around the mouth of your child and will extract the saliva from the cotton using a needleless syringe. This procedure can be repeated using new clean cotton swabs until at least 0.5mL of saliva is collected.

3. Consumption of sugar solution (for L/R test)

   After the baseline breath and saliva sample is collected, your child will be asked to consume about 10 mL solution containing the two types of sugars - lactulose (1g) and rhamnose (0.2g).

4. Heavy water dose administration.

   After the sugar dose, your child will be drinking a dose of heavy water from a needleless syringe.

   The heavy water dose is like the usual water that we drink, and it is naturally abundant in our body at a lower amount. The dose that will be given to your child is only about 1mL. It is safe and does not cause any harm to your child. We have been using this technique in adults and children for years now and no unwanted effects have been recorded since.

5. Sucrose dose administration.

   Your child will then consume a labelled sucrose dose (10µL of 40 mg/mL per kg body weight) in a total of 3mL water. This has the same composition as the common sugar. There are no harmful consequences of taking the dose and the test has been used in previous studies.

6. Post-dose sample collection.

   After consumption of the sucrose, breath will be collected every 15 minutes for the first hour and 30 minutes after until the 4th hour. A total of 10 post-dose breath samples will be collected.

   Your child's urine will be collected starting from 30-minute after the L-R sugar solution consumption until the 5th hour. The total volume will be recorded, and a portion will be stored. A pre-weighed urine bag with cotton will be used.

   Saliva samples will also be collected again 3 hours after the dose administration.

On your waiting time, we will be assessing:

1. Your socioeconomic status. Questions include water and sanitation, household assets, maternal education, and household income (WAMI index questionnaire).
2. The foods and beverages that your child consumed as reflected in the food diary. Details include the type and amount of food eaten, the method of preparation, the time the food is taken, and the duration of breastfeeding, if any.
3. Anthropometry of your child. Arm circumference, head circumference, and skinfold thickness will be measured by a trained project staff.

Your child is also required to submit a baseline stool sample (thumb-size). During your visits, we will monitor when the child poops.

Visit 1 lasts for about 5 hours. Snack and meals will be provided.

Study Visit 2: Dual Stable Isotope Tracer (DSIT)

One test will be conducted on the following day at FNRI. Your child needs to be in fasting state, that is he/she should not eat or drink any food or breastmilk for 1 hour before the test. Samples to be collected are breath, and blood. The steps include:

1. Baseline sample collection.

   One breath sample will be collected as previously described.

   A 4mL of blood sample will be extracted from your child by a registered medical technologist.

2. Consumption of mini-meal (DSIT technique)

   A complementary food (rice-mongo) containing stable isotope (deuterated amino acid and C-13 labelled spirulina) will be given to your child in small portions which will he/she needs to consume after the baseline collection and every hour for 5 hours. The first mini-meal is three times the portion of the succeeding mini-meal. Your child must consume the first meal within 15 minutes.

Like sucrose, the isotope labelled amino acid, and spirulina is harmless and safe to consume like unlabeled ones. Spirulina is a non-toxic, blue-green algae that is usually consumed by humans because of its nutritional and health benefits. Amino acids are compositions of protein. In this current study, its nutritional effects are not being studied, and it is used only as a way to assess your child's digestive capacity.

3. <u>Post-administration sample collection.</u>

   Three breath samples will be collected at 5, 5.5, and 6h after consumption of the first mini-meal.

   One blood sample (2mL) will also be extracted at 6h.

Visit 2 lasts for about 6-7hrs. Snack and meals will be provided.

Study Visit 3: Day 1 of Intervention

This visit is the start of the intervention. We will give your child a complementary food with or without the supplement depending on the group he/she is randomly assigned to. On this visit, we will demonstrate how to prepare the assigned food which your child must eat daily for 28 consecutive days.

The complementary food is an instant rice-mongo blend developed by DOST-FNRI. It is a powder mix and can be prepared by adding hot water.

The supplement is a mixture of essential amino acids. Only the intervention group will be given the supplement. It will be added to the rice-mongo meal or into other complementary food you wish to give your child, and it must be recorded.

On scheduled days for at least 3x a week, we will be observing your food preparation and the feeding session of your child. Visits 3 and 4 are part of the observed feeding sessions that will be conducted at FNRI. Other times, we will be the one going to your house for the observation.

The complementary food will be ideally given as a first meal of the day. This is just a part of the child's food daily intake. You are free to plan and prepare the foods your child will take on the rest of the day.

Visit 3 lasts for about an hour. Snack and meals will be provided.

Observed Feeding Sessions

For three times a week, a study staff will visit you at your house or a nearby barangay center and observe the feeding. We will remind you of our visit. Also, we will:

1. Check your child's vital signs (body temperature, blood pressure, pulse rate and oxygen status)
2. Ask you if your child got sick or something unusual happened
3. Ask you whether your child consumed the given complementary food (with or without the supplement)
4. Ask you about the foods and beverages that your child consumed for the past three days other than the complementary food as reflected in the food diary.

Every visit lasts for about 1 hr.

Follow-up Visits: Endline tests

After the 28-day intervention period, you and your child will visit FNRI for another round of tests that is described above:

1. SBT and L/R will be done a day after the last day of intervention. Details are discussed in Study Visit 1
2. DSIT will be done at the second day after. Details are discussed in Study Visit 2.
3. Collection of stool sample (thumb-size)

After 6 months, we will follow you up and conduct the following:

4. Anthropometric measurement
5. Body composition measurement using DDT. Details are discussed in Study Visit 4 except that the complementary food will not be given to your child.
6. Ask you about your child's food intake in the past 24 hours (24-hour food recall).

DURATION OF PARTICIPATION AND RETENTION OF SAMPLES

In general, the study duration will be about 7 months in total including the baseline assessment, the 28-day intervention, and the endline assessment. A calendar showing the assessments to be taken will be provided.

If there are no delays due to adverse events, the intervention will continue for 28 days or until one of the following happens:

- Your child is not able to tolerate the supplement (throws up)
- Your child is diagnosed with severe acute malnutrition (SAM)
- Your child has illness that prevents further administration of treatment
- Unacceptable adverse event/s
- You voluntarily withdraw your child from the treatment
- You and your child are not able to comply with the protocol requirements
- You and your child cannot be located or not responding to our calls
- Changes in the child's condition that upon our judgment might be better to stop his/her participation

Your child's anonymized biological samples (stool, breath, plasma, saliva, and urine) will be kept for 5 years or until they are used up. By signing this form, you are also allowing us to use the samples for future use as in-house sample for method validation or part of a study that is related to malnutrition or intestinal conditions. Stored samples will be analyzed within the mentioned scope. You may also object or withhold consent if ever there are diversions from what have been discussed here. All unconsumed samples will be disposed properly after 5 years.

RISKS

Complementary foods and the supplement to be consumed will be handled with safety precautions and will be prepared using food-grade materials. The supplement is nutritional, and dosages of IAA in the range used in the intervention are rarely associated with reported side effects.

There are minimal risks and discomforts during collection of biological samples (breath, stool, urine, blood and saliva) and consumption of doses and supplements. Your child may feel discomfort when blowing through a straw/tube/pediatric mask during breath collection, when the urine bag will be placed in his/her genital area during urine collection, and when cotton swabbing around his/her mouth during saliva collection. These procedures are necessary, and we will make sure that we will do this with your supervision and at most safe way possible. Your child may also feel pain during blood extraction and may have a small bruise. This is normal and the bruise should quickly go away. In case a small bruise is formed, a cold compress will be used to help it subside within the first 24 hours and a warm compress will be used after. All materials that will be used are sterile and safe.

You are responsible to report to us any untoward events that happens to your child during he/she is enrolled as a study participant. We, together with a pediatrician, will assess the severity of the event and investigate if it is related to the study. In case of study-related injury, we will provide you with first aid or necessary medical assistance as emergency arises. We will do follow-ups until an unacceptable event is resolved.

BENEFITS AND COMPENSATION

Both complementary foods and supplements to be given contain nutrients that contribute to the proper growth and development of infants. However, this study is purely research and there is no guarantee that your child's health and nutritional status will improve after the intervention. By joining in the study, you are helping us to get evidence that will provide scientific basis for the development of nutritional/health intervention and policies as well as better opportunities to help reduce stunting in children.

Aside from free foods, your child will also get a free medical consultation during the screening process. Every visit at FNRI, you will receive a cash incentive and reimbursement of your transportation expenses. Load allowance will also be given on Visit 1 and on the 28th day of intervention for better communication.

In case of study-related injury, you will receive a compensation for the expenses used for hospitalization, transportation, and necessary treatment/medicines.

CONFIDENTIALITY

All the information that will be gathered from you will remain confidential. A unique code will be assigned for every mother-child pair participant that will conceal your and your child's identity in all the forms/records that will be used and compiled in this study. Your name and your child's name will not appear in any project report

or publication. Hard copies of study files will be stored in a locked file cabinet and access will only be limited to project team members. Electronic study files will be saved in a password-protected office computer.

SHARING THE RESULTS

You will be informed about your child's anthropometric measurements and the results of the doctor's assessment during the screening. In case of medical findings, you will be referred to have further consultation.

Since the analysis of the samples take time, we will not be able to give you feedback on the results of the tests. But the results will be published in scientific/academic publications. It may be presented in research forums, symposia, and other related activities, in order that other interested researchers and the general population may gain insights from this study. Moreover, the results will be pooled together with that from other participating countries. It will be ensured that your and your child's identity will remain confidential when the study is presented to the public.

WHO TO CONTACT

Should you have any question regarding the conduct of this study, please feel free to contact any member of the project team at tel. no. 8837-8113 local 341, or through mobile no. 09913601216 (Amster Fei P. Baquiran, Senior Science Research Specialist). The protocol of the study has been reviewed and approved by the FNRI Institutional Ethics Review Committee (FIERC), which is a committee whose task is to make sure that research participants are protected from harm. You may contact Marilita A. Aguilos at 8837-2071 loc. 2297 should you wish to find out more about the FIERC and the rights of the participants.

PART II: CERTIFICATE OF CONSENT

I have read or it has been read to me the information described above. I have had the opportunity to ask questions about it and any questions I asked have been answered to my satisfaction. I consent voluntarily for my child and I to participate in the study including:

1. Collection of information about you and your child (personal, demographic, anthropometric, food intake)
2. Collection of biological samples (breath, urine, stool, blood and saliva) from your child
3. Oral administration of solution or meal that contain sugars and/or stable isotope tracers for your child
4. Going to FNRI (you and your child) and house visits by study staff
5. Daily consumption of complementary food with or without supplement for 28 days
6. Taking of photos (which may include mother and the child) during the conduct of the research

I also give consent to the use and retention of the collected samples including their future use.

Name and signature of the Mother: ______________________________Date signed: ____________

Name and signature of the Father: ______________________________ Date signed: ____________

I have witnessed the orientation and reading of the consent form to the potential participant. The individual had the opportunity to ask questions and all have been answered. I confirm that the individual has given consent freely. A copy of this Informed Consent Form has been provided to the participant.

Name and signature of project staff: ______________________________ Date signed: ____________

The efficacy of amino acid supplementation in treating Zambian children with Environmental Enteropathy and stunting (AMAZE 2)

INFRMATION SHEET AND CONSENT FORM FOR CONTROLS

Principal Investigators: Dr Beatrice Amadi and Professor Paul Kelly

INTRODUCTION

We are researchers from TROPGAN and the University of Zambia School of Medicine. We would like to invite you and your child to be part of this research project. You should only agree that your child could take part if you want to; it is entirely up to you. Choosing not to take part will not affect your child's treatment in any way, now or in the future. Please read or listen to the following information carefully before you decide that your child can take part. If you decide to take part, you will be asked to sign a consent form to record your agreement. You are free to withdraw at any time during your child's participation, even without giving a reason.

WHY ARE WE DOING THIS TRIAL

The purpose of this trial is to develop extra treatments to help in treatment of stunting, which means poor height growth. We know that many children in this area, especially children who are not growing well, have problems in the gut (intestine), which can allow it to become 'leaky'. We believe that if this does not heal properly, children do not grow properly. We want to try giving additional food to heal the damaged intestine.

WHY HAS MY CHILD BEEN CHOSEN?

We have identified that your child is growing very well. We need to study some healthy children to allow us to identify correctly the problems which are preventing good growth in the other children.

WHAT THE STUDY INVOLVES

We think that giving additional amino acids may help growth and we want to compare this intervention against the treatments we currently use. We do not know if this will work better than the treatments we already have so we need to compare them in a randomised clinical trial. This means that the choice of treatments is made randomly, by chance (like the flip of a coin). We also need to study children who are growing well to allow us to spot the differences.

THE INVESTIGATIONS INVOLVED

When your child enters the study, he/she will have a full medical assessment and examination if he or she has any problems. All these tests are completely safe and we will pay for them. For our research, we will also ask for blood samples, stool samples, urine samples, and saliva samples. The total volume of blood we will collect for research is limited to 5ml. We will carry out HIV testing as part of the tests on blood samples; the results will be completely confidential even if it is negative.

There will be one day of assessments. We will ask for a stool sample and we will collect two blood samples (3ml and 2ml – we will show you what this looks like), a saliva sample, and a urine collection over 3 hours after giving a sugar solution, and we will collect four

breath samples. At four timepoints we will also put a finger clip on one finger to measure fluorescence. We will show you an example of what this looks like; it is completely painless.

If you would like, we would be very happy to show around St Augustine Clinic, so you have a better idea of what is involved. We can also show you the laboratories so you can see what we do with the samples we take, and how we analyse them.

HOW MANY CHILDREN WILL BE IN THE STUDY
We will study 60 children with stunting, and we will also study 30 children who do not have stunting.

WHAT DO WE DO WITH YOUR CHILD'S SAMPLES
We only use the samples you provide for research. Any samples that we do not need for research will be destroyed. We have no need to use the samples you provide for any other purpose, including giving them to people who are not involved in the research. It is also against the law to do so.

We do nearly all of the research analysis in the laboratories at St Augustine Clinic. Some of the tests will need to be done by our collaborators in the UK, so some of your child's samples may be sent there. These collaborating laboratories are not allowed to do anything with the samples that isn't to do with research, including selling them or giving them to people who aren't involved in the research. We are very strict on this and Dr Beatrice Amadi can give you absolute reassurance that the samples are only used for this research. Once the research is finished we will archive any remaining samples in our biobank which is licenced by the National Health Research Authority.

We do many different measurements on the samples you provide, and if you are interested we can demonstrate and explain some of these to you, including how the samples are stored.

WHAT DO WE DO WITH THE INFORMATION YOU PROVIDE
During the research we collect information about you and your child. We store the information on computers and remove your name and your child's name and any other ways of being identified, so that there is no way of telling who the information refers to. Any and all information you give us is confidential. This means that we are not allowed to tell anyone about you or your child, even if they are friends or family.
Note: Should there be any new information that we discover as the study goes on that might affect your child, we will not hesitate to inform you.

HOW THIS STUDY MIGHT BENEFIT YOU
We will begin by giving your child a thorough clinical assessment. If any specific treatment is required we will provide that.

We hope that our learning from this trial will benefit children in the future. We will hold a caregiver meeting at the end of the trial to inform you of the results.

WHAT ARE THE COSTS TO ME/MY CHILD?
There is no payment for participating in this trial. We will assist you with transport money to return home.

ARE THERE ANY RISKS TO PARTICIPATING IN THIS STUDY?
This is a research project, so there is the discomfort of collecting samples.

WHAT IF I DON'T WANT TO PARTICIPATE IN THE STUDY OR LATER DECIDE TO WITHDRAW?
Participation in this study is voluntary. It is completely up to you whether or not your child participates, or if you join the trial but decide to withdraw later. If you decide for your child not to participate, it will not affect the treatment your child receives now or in the future. Whatever your decision, it will not affect your relationship with the staff caring for you.

INSURANCE AND WHAT TO DO IF YOU HAVE QUESTIONS OR CONCERNS
In the unlikely event that your child is harmed as a result of this trial, we have insurance to cover any harms. These arrangements do not affect your right to pursue a claim through legal action.

It is up to you to decide whether or not to take part. If you do decide to take part you will be given this information sheet to keep and be asked to sign a consent record form.
Note: You might be asked to come back for follow up tests after your child has been discharged. Should this happen, we will ensure that we make transport arrangements. We will arrange our driver to pick you up and drop you off and if this will not be possible, we will make other transport arrangements for you to ensure that you do not spend any money to this effect.

If you have any questions or concerns about the study or how it is conducted, you can contact Professor Kelly's and Dr Amadi's team in the hospital at any point.

Contact details are:

Professor Paul Kelly or Dr Beatrice Amadi
St Augustine Clinic
26 Esther Lungu Road
Kamwala South
Lusaka
0953 371260 or 0969 110587

If you are not happy to do this, or are not happy with the advice you get from Professor Kelly's and Dr Amadi's team, you can contact:
The University of Zambia Biomedical Research Ethics Committee,
University of Zambia School of Medicine,
Ridgeway Campus,
PO Box 50110,
Lusaka.
tel: 0211 250753 and speak to Lorna

SID GT |__|__|__|__|__| TID GT |__|__|__|__|__|

CONSENT RECORD

Please complete this form after you have read or listened to the Information Sheet and/or listened to an explanation about the research.

1. I have read or listened to the Participant Information Sheet for this study (version 0.1, dated 9th May 2024).
2. The research has been explained to me. I understand what the research involves and what is required of me.
3. I understand that my child will have a blood test for HIV.
4. I understand that samples obtained from my child may be sent abroad for research purposes only, and that after use the samples will either be returned to Zambia for storage or destroyed.
5. I consent to the long term storage of any samples I provide (including blood, urine, stool, small bowel fluid and endoscopic biopsies) in secure facilities at St Augustine after this research study has finished, for ethically approved research projects on malnutrition.
6. I understand that there is no obligation for me to allow my child to participate in the research study, and that I do not have to give a reason for not participating.
7. I understand that if I decide at any time during the research that I no longer wish my child to participate, I can notify the researchers involved and be withdrawn from it immediately, without giving a reason.
8. I consent to the processing of our personal information for the purposes of this research study. I understand that such information will be treated as strictly confidential at all times and that any identifiable information about me or my child will be available only to members of the study team.

Participant's Statement:

I ____ have read or listened to statements 1-8 above and I agree to allow my child _____________________________________to take part in the study.

Signed: Date:

Thumb Print: Date

Witnessed: Date:

Thumb Print: Date:

Investigator's Statement:

I ____ confirm that I have carefully explained the nature, demands and any foreseeable risks of the proposed research to the volunteer.

Signed: Date:

COPIES MUST BE KEPT IN THE CONSENT REGISTER AND GIVEN TO THE PARENT/CARER

The efficacy of amino acid supplementation in treating Zambian children with Environmental Enteropathy and stunting (AMAZE 2)

INFRMATION SHEET AND CONSENT FORM

Principal Investigators: Dr Beatrice Amadi and Professor Paul Kelly

INTRODUCTION

We are researchers from TROPGAN and the University of Zambia School of Medicine. We would like to invite you and your child to be part of this research project. You should only agree that your child could take part if you want to; it is entirely up to you. Choosing not to take part will not affect your child's treatment in any way, now or in the future. Please read or listen to the following information carefully before you decide that your child can take part. If you decide to take part, you will be asked to sign a consent form to record your agreement. You are free to withdraw at any time during your child's participation, even without giving a reason.

WHY ARE WE DOING THIS TRIAL

The purpose of this trial is to develop extra treatments to help in treatment of stunting, which means poor height growth. We know that many children in this area, especially children who are not growing well, have problems in the gut (intestine), which can allow it to become 'leaky'. We believe that if this does not heal properly, children do not grow properly. We want to try giving additional food to heal the damaged intestine.

WHY HAS MY CHILD BEEN CHOSEN?

We have identified that your child has not been growing as fast as we would like. We want to include your child in a trial of stunting that will include additional amino acids alongside the standard of care.

WHAT THE STUDY INVOLVES

We think that giving additional amino acids may help growth and we want to compare this intervention against the treatments we currently use. We do not know if this will work better than the treatments we already have so we need to compare them in a randomised clinical trial. This means that the choice of treatments is made randomly, by chance (like the flip of a coin).

A set of envelopes has been prepared with numbers on. If you agree that your child is to be included in the trial, a study number will be given. Once that number has been given we will open the envelope in your presence with that number and see whether your child should be given the additional amino acids, or the standard care only. It is very important that you understand these two principles of a randomised trial:

1. We genuinely do not know if amino acids will help when given alongside the treatment that is already being given.
2. Children will be allocated a treatment by chance.

Once the envelope has been opened, we will follow the treatment course indicated on the contents of the envelope. We will **not** choose the treatment for your child.

AMINO ACID TREATMENT

To grow, children need protein, and proteins are made of amino acids. Lots of trials have been done in which children with stunting have been given extra foods, but here we want to try if giving just particular amino acids might be more useful. Body builders use these amino acids, but that's a very different situation to the growth of a young child so we cannot predict whether it will help. The amino acids are given in the form of a powder which will be mixed with orange juice, and the children who are not getting the amino acids will just get a daily drink of the orange juice.

THE INVESTIGATIONS INVOLVED

When your child enters the study, he/she will have a full medical assessment and examination to see if we can identify a cause for their poor growth. All these tests are completely safe and we will pay for them. For our research, we will also ask for blood samples, stool samples, urine samples, and saliva samples, and at the end of the period of treatment your child will have an endoscopy. The total volume of blood we will collect for research is limited to a safe quantity (2ml/kg body weight in total during the trial). We will carry out HIV testing as part of the tests on blood samples; the results will be completely confidential even if it is negative. All children will be monitored frequently by our team of doctors and nurses for a full four weeks.

There will be three days of full assessments. Before starting the daily orange juice drink and at the end of the period of treatment (4 weeks) we will ask for a stool sample and there will be a full day of collecting samples: two blood samples (3ml and 2ml – we will show you what this looks like), a saliva sample, and a urine collection over 3 hours after giving a sugar solution, and we will collect four breath samples. At four timepoints we will also put a finger clip on one finger to measure fluorescence. We will show you an example of what this looks like; it is completely painless.

In addition, on the last day we will carry out an endoscopy. This means examining the inside of the stomach and intestine with a small flexible tube, through which we can collect tiny pieces of the lining of the intestine, each about the size of the head of a match. Although this sounds rather worrying, it is quite safe because we have good facilities and a great deal of experience. The endoscopy requires giving anaesthetic injections; this is done by an anaesthetist who monitors the children very carefully. If you would like, we would be very happy to show you the endoscopy unit and equipment in St Augustine Clinic, so you have a better idea of what is involved. We can also show you the laboratories so you can see what we do with the samples we take, and how we analyse them.

HOW MANY CHILDREN WILL BE IN THE STUDY

We will study 60 children, and we will also study 30 children who do not have stunting.

WHAT DO WE DO WITH YOUR CHILD'S SAMPLES

We only use the samples you provide for research. Any samples that we do not need for research will be destroyed. We have no need to use the samples you provide for any other purpose, including giving them to people who are not involved in the research. It is also against the law to do so.

We do nearly all of the research analysis in the laboratories at St Augustine Clinic. Some of the tests will need to be done by our collaborators in the UK, so some of your child's samples may be sent there. These collaborating laboratories are not allowed to do anything with the samples that isn't to do with research, including selling them or giving them to people who aren't involved in the research. We are very strict on this and Dr Beatrice Amadi can give you absolute reassurance that the samples are only used for this research. Once the research is finished we will archive any remaining samples in our biobank which is licenced by the National Health Research Authority.

We do many different measurements on the samples you provide, and if you are interested we can demonstrate and explain some of these to you, including how the samples are stored.

WHAT DO WE DO WITH THE INFORMATION YOU PROVIDE

During the research we collect information about you and your child. We store the information on computers and remove your name and your child's name and any other ways of being identified, so that there is no way of telling who the information refers to. Any and all information you give us is confidential. This means that we are not allowed to tell anyone about you or your child, even if they are friends or family.
Note: Should there be any new information that we discover as the study goes on that might affect your child, we will not hesitate to inform you.

HOW THIS STUDY MIGHT BENEFIT YOU

We will begin by searching thoroughly for anything beyond the gut which might be contributing to your child's poor growth. This may allow us to provide specific treatment. Your child will also get a daily drink of orange juice, which is tasty. At the end of the trial the endoscopy may provide additional information which may benefit your child; this has helped several children in previous studies. We hope that additional amino acids will speed up your child's growth. However, we cannot guarantee this because we genuinely do not know if they will help. Your child will continue to receive the best possible care for malnutrition as well as any new treatments we give.

We hope that our learning from this trial will also benefit children with stunting in the future. We will hold a caregiver meeting at the end of the trial to inform you of the results.

WHAT ARE THE COSTS TO ME/MY CHILD?

There is no payment for participating in this trial. We will assist you with transport money to return home and for you to bring back your child for the 28 days of the study. We will cover the costs of any additional tests or treatment your child may need to help with their growth.

ARE THERE ANY RISKS TO PARTICIPATING IN THIS STUDY?

This is a research project, and while we think these amino acids will help we cannot absolutely guarantee that there will be no unwanted effects from giving these higher doses of amino acids. We have considered this carefully and done our best, but the possibility remains that some children might experience adverse reactions to these treatments. We have ensured that we have trained our staff to look out for these side effects and when it does happen we will provide the best care to ensure that your child is okay.

WHAT IF I DON'T WANT TO PARTICIPATE IN THE STUDY OR LATER DECIDE TO WITHDRAW?
Participation in this study is voluntary. It is completely up to you whether or not your child participates, or if you join the trial but decide to withdraw later. If you decide for your child not to participate, it will not affect the treatment your child receives now or in the future. Whatever your decision, it will not affect your relationship with the staff caring for you.

INSURANCE AND WHAT TO DO IF YOU HAVE QUESTIONS OR CONCERNS
In the unlikely event that your child is harmed as a result of this trial, we have insurance to cover any harms. These arrangements do not affect your right to pursue a claim through legal action.

It is up to you to decide whether or not to take part. If you do decide to take part you will be given this information sheet to keep and be asked to sign a consent record form.
Note: You might be asked to come back for follow up tests after your child has been discharged. Should this happen, we will ensure that we make transport arrangements. We will arrange our driver to pick you up and drop you off and if this will not be possible, we will make other transport arrangements for you to ensure that you do not spend any money to this effect.

If you have any questions or concerns about the study or how it is conducted, you can contact Professor Kelly's and Dr Amadi's team in the hospital at any point.

Contact details are:

Professor Paul Kelly or Dr Beatrice Amadi
St Augustine Clinic
26 Esther Lungu Road
Kamwala South
Lusaka
0953 371260 or 0969 110587

If you are not happy to do this, or are not happy with the advice you get from Professor Kelly's and Dr Amadi's team, you can contact:
The University of Zambia Biomedical Research Ethics Committee,
University of Zambia School of Medicine,
Ridgeway Campus,
PO Box 50110,
Lusaka.
tel: 0211 250753 and speak to Lorna

SID GT |__|__|__|__|__| TID GT |__|__|__|__|__|

CONSENT RECORD

Please complete this form after you have read or listened to the Information Sheet and/or listened to an explanation about the research.

1. I have read or listened to the Participant Information Sheet for this study (version 0.1, dated 9th May 2024).
2. The research has been explained to me. I understand what the research involves and what is required of me.
3. I understand that my child will have a blood test for HIV.
4. I understand that samples obtained from my child may be sent abroad for research purposes only, and that after use the samples will either be returned to Zambia for storage or destroyed.
5. I consent to the long term storage of any samples I provide (including blood, urine, stool, small bowel fluid and endoscopic biopsies) in secure facilities at St Augustine after this research study has finished, for ethically approved research projects on malnutrition.
6. I understand that there is no obligation for me to allow my child to participate in the research study, and that I do not have to give a reason for not participating.
7. I understand that if I decide at any time during the research that I no longer wish my child to participate, I can notify the researchers involved and be withdrawn from it immediately, without giving a reason.
8. I consent to the processing of our personal information for the purposes of this research study. I understand that such information will be treated as strictly confidential at all times and that any identifiable information about me or my child will be available only to members of the study team.

Participant's Statement:
I ______________________________________ have read or listened to statements 1-8 above and I agree to allow my child _________________________________to take part in the study.

Signed: Date:
Thumb Print: Date

Witnessed: Date:
Thumb Print: Date:

Investigator's Statement:

I ______________________________________ confirm that I have carefully explained the nature, demands and any foreseeable risks of the proposed research to the volunteer.

Signed: Date:

COPIES MUST BE KEPT IN THE CONSENT REGISTER AND GIVEN TO THE PARENT/CARER
