# Supplementary material for: Impact of indispensable amino acid supplementation on gut function in children at high risk of environmental enteropathy: protocol for an international coordinated group of randomised controlled trials
Source: BMJ Open. 2026 Apr 24;16(4):e105456. doi: 10.1136/bmjopen-2025-105456 (PMC13110547; doi:10.1136/bmjopen-2025-105456)
Supplement: online supplemental file 3 [file bmjopen-16-4-s003.docx]

**SUPPLEMENT 2. Details of Intervention (IAA Supplement Mix)**

The IAA supplement is a nutritional supplement developed by the Study Investigators using WHO/FAO guidelines on IAA requirements for children ([Protein and amino acid requirements in human nutrition : report of a joint FAO/WHO/UNU expert consultation](https://iris.who.int/items/78c03092-bcc2-40f5-90d1-60641ecdf6ea). ISBN 9241209356. https://iris.who.int/handle/10665/43411). Each daily dose of the supplement was designed to provide 1.5 x estimated average requirements for each child. The supplement was produced by Northman Nutrition Limited, UK for the study. The IAA supplement is a white to off-white, finely granulated crystalline powder composed of nine IAAs—L-Histidine, L-Isoleucine, L-Leucine, L-Lysine HCl, L-Methionine, L-Phenylalanine, L-Threonine, L-Tryptophan, and L-Valine—augmented with glycine to promote gastrointestinal repair. Raw materials arrived in tamper-evident containers with CoAs, stored at 5–20°C, <50% RH, Quarantined for incoming quality control via HPLC, ICP-MS, and visual inspection, weighed in an ISO 8 cleanroom using calibrated balances, blended to ensure uniformity, with nitrogen purging to prevent oxidation, sieved to remove oversize particles, filled in sachets and heat-sealed, and then inspected for seal integrity and weight verification. The mix is packaged in 1kg heat-sealed, triple-layer foil pouches (polyethylene/aluminum/polyester, 100 µm thickness), ensuring a moisture vapor transmission rate (MVTR) of <0.5 g/m²/day at 60% RH. The mixture is stable for 2 years based on 6-month data (no degradation observed). A certificate of analysis was supplied by Nutraceuticals Group Europe (Batch NIGEBLE018060a) for the following parameters: *Heavy Metals:*Pb <0.005 mg/kg, As <0.002 mg/kg, Cd <0.001 mg/kg. *Microbiology:* Total Plate Count 20 cfu/g, E. coli absent, Salmonella absent. *Purity:* All EAAs ≥99.2% (HPLC). CoAs comply with USP/Ph. Eur. and SADC food-grade standards.
