# Supplementary material for: Impact of indispensable amino acid supplementation on gut function in children at high risk of environmental enteropathy: protocol for an international coordinated group of randomised controlled trials
Source: BMJ Open. 2026 Apr 24;16(4):e105456. doi: 10.1136/bmjopen-2025-105456 (PMC13110547; doi:10.1136/bmjopen-2025-105456)
Supplement: Supplementary file 4 [file bmjopen-2025-105456.reviewer_comments.pdf]

PEER REVIEW HISTORY

BMJ Open publishes all reviews undertaken for accepted manuscripts. Reviewers are asked to complete a checklist review form and are provided with free text boxes to elaborate on their assessment. These free text comments are reproduced below.

ARTICLE DETAILS

Title (Provisional)

Impact of indispensable amino acid supplementation on gut function in children at high risk of environmental enteropathy: protocol for an international coordinated group of randomized controlled trials

Authors

Lee, Gwenyth O; Owino, Victor; Baquiran, Amster Fei P.; Pasanna, Roshini M; Achoribo, Seyram Elom; Meskini, Toufik; Amadi, Beatrice; Maleta, Kenneth Mphatso; Gaudichon, Claire; Serafico, Michael E.; Hegde, Shalini; Cabanilla, Carl Vincent D; Devi, Sarita; El Mzibri, Mohammed; Brouwer, Andrew F; Kurpad, Anura V; Kelly, Paul; Morrison, Douglas

---

VERSION 1 - REVIEW

Reviewer 1

Name Huo, Junsheng

Affiliation National Institute for Nutrition and Health, China CDC, Nutrition and Health

Date 05-Oct-2025

COI None

This study aims to observe the necessity of supplementing indispensable amino acids for children aged 18 to 36 months with high risk of environmental enteropathy through a multi-center research in six countries. This study protocol is very well designed and described. It is expected that the study will achieve its main and secondary targets, which will be highly valuable for public strategy in prevention of those at risk of development of gut function induced by indispensable amino acids.

As described in study intervention in the manuscript, the intervention designed in this study consists of two ways: providing standard complementary foods to the control group and providing standard complementary foods along with IAA supplements to the intervention group. The standard complementary foods will be developed by the project working group in the respective countries, with main foods including rice, eggs, yogurt, beans, and others. The IAA supplements used in this study were of a uniform formula and manufactured in a same

batch. However, as a key aspect of the study, more information of the standard complementary foods and the IAA supplement should be needed. Since the detailed facts may need to complete during implementation, if possible, the basic principles and methods for formulating the standard complementary foods should be provided to ensure that the pattern of standard complementary foods and the amounts of nutrients supplied, especially protein derived IAA intakes, can match those of the study groups, thereby avoiding the possibility of IAA intake reaching a level from standard complementary food provided interfering on the observation results of the trial. A more detailed description of the IAA supplements should also be need if possible, including requirements of quality specifications, raw material, producers, quality assurance systems, and inspection requirements.

VERSION 1 - AUTHOR RESPONSE

Reviewer: 1
Prof. Junsheng Huo, National Institute for Nutrition and Health, China CDC
Comments to the Author:
This study aims to observe the necessity of supplementing indispensable amino acids for children aged 18 to 36 months with high risk of environmental enteropathy through a multi-center research in six countries. This study protocol is very well designed and described. It is expected that the study will achieve its main and secondary targets, which will be highly valuable for public strategy in prevention of those at risk of development of gut function induced by indispensable amino acids.
RESPONSE. We thank the reviewer for their positive appraisal of the study, as well as their constructive feedback.

As described in study intervention in the manuscript, the intervention designed in this study consists of two ways: providing standard complementary foods to the control group and providing standard complementary foods along with IAA supplements to the intervention group. The standard complementary foods will be developed by the project working group in the respective countries, with main foods including rice, eggs, yogurt, beans, and others. The IAA supplements used in this study were of a uniform formula and manufactured in a same batch. However, as a key aspect of the study, more information of the standard complementary foods and the IAA supplement should be needed. Since the detailed facts may need to complete during implementation, if possible, the basic principles and methods for formulating the standard complementary foods should be provided to ensure that the pattern of standard complementary foods and the amounts of nutrients supplied, especially protein derived IAA intakes, can match those of the study groups, thereby avoiding the possibility of IAA intake reaching a level from standard complementary food provided interfering on the observation results of the trial. A more detailed description of the IAA supplements should also be need if possible, including requirements of quality specifications, raw material, producers, quality assurance systems, and inspection requirements.
RESPONSE. We have clarified this within the text. The study teams will not prescribe specific complementary foods to participants during the intervention period. Instead, families/caregivers participating in the study are asked to follow habitual practices (providing habitual complementary foods), either with (intervention group) or without (control group) the IAA supplement. We do expect variation between sites in the IAA composition of these

foods. To evaluate this, samples of the habitual foods consumed by children during the study will be collected, and their IAA score determined by standard methods to give an estimate of the foods consumed. This will be reported in the study results.
We have also added the following more detailed description of the IAA supplement to the supplemental materials (Supplement 2):
The IAA supplement is a nutritional supplement developed by the Study Investigators using WHO/FAO guidelines on IAA requirements for children (Protein and amino acid requirements in human nutrition : report of a joint FAO/WHO/UNU expert consultation. ISBN 9241209356. https://iris.who.int/handle/10665/43411). Each daily dose of the supplement was designed to provide 1.5 x estimated average requirements for each child. The supplement was produced by Northman Nutrition Limited, UK for the study. The IAA supplement is a white to off-white, finely granulated crystalline powder composed of nine IAAs—L-Histidine, L-Isoleucine, L-Leucine, L-Lysine HCl, L-Methionine, L-Phenylalanine, L-Threonine, L-Tryptophan, and L-Valine—augmented with glycine to promote gastrointestinal repair. Raw materials arrived in tamper-evident containers with CoAs, stored at 5–20°C, <50% RH, Quarantined for incoming quality control via HPLC, ICP-MS, and visual inspection, weighed in an ISO 8 cleanroom using calibrated balances, blended to ensure uniformity, with nitrogen purging to prevent oxidation, sieved to remove oversize particles, filled in sachets and heat-sealed, and then inspected for seal integrity and weight verification. The mix is packaged in 1kg heat-sealed, triple-layer foil pouches (polyethylene/aluminum/polyester, 100 µm thickness), ensuring a moisture vapor transmission rate (MVTR) of <0.5 $g/m^2/day$ at 60% RH. The mixture is stable for 2 years based on 6-month data (no degradation observed). A certificate of analysis was supplied by Nutraceuticals Group Europe (Batch NIGEBLE018060a) for the following parameters: Heavy Metals: Pb <0.005 mg/kg, As <0.002 mg/kg, Cd <0.001 mg/kg. Microbiology: Total Plate Count 20 cfu/g, E. coli absent, Salmonella absent. Purity: All EAAs ≥99.2% (HPLC). CoAs comply with USP/Ph. Eur. and SADC food-grade standards.

Reviewer: 1
If you have selected ‘Yes’ above, please provide details of any competing interests.: Not applicable.
